# Evaluation of Nontargeted Mass Spectral Data Acquisition Strategies for Water Analysis and Toxicity-Based Feature Prioritization by MS2Tox

**DOI:** 10.1021/acs.est.4c02833

**Published:** 2024-09-19

**Authors:** Pilleriin Peets, May Britt Rian, Jonathan W. Martin, Anneli Kruve

**Affiliations:** † Department of Materials and Environmental Chemistry, Stockholm University, Svante Arrhenius Väg 16, SE-106 91, Stockholm, Sweden; ‡ Department of Environmental Science, Stockholm University, Svante Arrhenius Väg 16, SE-106 91 Stockholm, Sweden; § Institute of Biodiversity, Faculty of Biological Science, Cluster of Excellence Balance of the Microverse, Friedrich-Schiller-University Jena, 07743, Jena, Germany; ∥ National Facility for Exposomics, Metabolomics Platform, Science for Life Laboratory, Stockholm University, Solna 171 65, Sweden

**Keywords:** nontargeted analysis, nontargeted screening, high-resolution mass spectrometry, machine-learning, toxicity prediction, MS/MS data acquisition methods, LC-HRMS, LC_50_

## Abstract

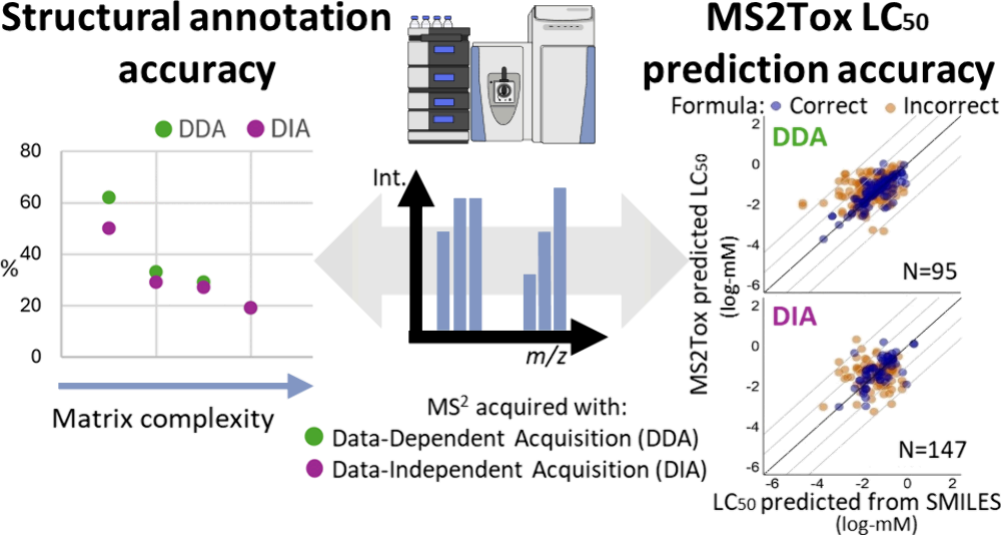

The machine-learning tool MS2Tox can prioritize hazardous nontargeted molecular features in environmental waters, by predicting acute fish lethality of unknown molecules based on their MS^2^ spectra, prior to structural annotation. It has yet to be investigated how the extent of molecular coverage, MS^2^ spectra quality, and toxicity prediction confidence depend on sample complexity and MS^2^ data acquisition strategies. We compared two common nontargeted MS^2^ acquisition strategies with liquid chromatography high-resolution mass spectrometry for structural annotation accuracy by SIRIUS+CSI:FingerID and MS2Tox toxicity prediction of 191 reference chemicals spiked to LC-MS water, groundwater, surface water, and wastewater. Data-dependent acquisition (DDA) resulted in higher rates (19–62%) of correct structural annotations among reference chemicals in all matrices except wastewaters, compared to data-independent acquisition (DIA, 19–50%). However, DIA resulted in higher MS^2^ detection rates (59–84% DIA, 37–82% DDA), leading to higher true positive rates for spectral library matching, 40–73% compared to 34–72%. DDA resulted in higher MS2Tox toxicity prediction accuracy than DIA, with root-mean-square errors of 0.62 and 0.71 log-mM, respectively. Given the importance of MS^2^ spectral quality, we introduce a “CombinedConfidence” score to convey relative confidence in MS2Tox predictions and apply this approach to prioritize potentially ecotoxic nontargeted features in environmental waters.

## Abstract 2

Two common MS^2^ data acquisition strategies for nontargeted analysis of water were compared with respect to molecular coverage, structural annotation, and performance in MS2Tox, a machine-learning tool for prediction of acute fish toxicity.

## Introduction

A wide variety of anthropogenic substances have been identified in groundwaters, surface waters, and wastewaters, including pharmaceuticals, personal care products, industrial additives, per- and polyfluoroalkyl substances (PFAS), and pesticides,^1−8^ with a range of toxicities to humans and wildlife.^9−11^ While some of these are priority substances that are routinely monitored according to national or international programs and directives, 95% of chemicals in commerce have never been monitored in the environment and may have multiple transformation products for which exposure and hazard have not yet been assessed.^12^ In fact, more than 370,000 chemicals or chemical mixtures are registered for production and use globally, many of which have unknown or ambiguous structures.^13^ Thus, new tools and approaches are needed to monitor chemical pollution that can identify unknown toxic substances in the environment.

Nontargeted analysis of environmental samples by liquid chromatography high-resolution mass spectrometry (LC-HRMS) has evolved as a powerful tool for acquiring comprehensive molecular information from environmental samples. Thousands, or even tens of thousands, of unknown molecular features can be detected in environmental samples,^14,15^ with each feature having a chromatographic retention time and peak area(s), full scan mass spectrum (i.e., MS^1^), and possibly a fragmentation spectrum (i.e., MS^2^).^16,17^ Structural annotation of unknown features can to some extent be accomplished by MS^2^ spectral matching to reference libraries (e.g., MassBank^18^), or use of *in silico* structural annotation tools.^19,20^ In both cases the annotation rate and accuracy depend on the availability of reference MS^2^ spectra, as well as the quality of acquired MS^2^. Even with quality experimental MS^2^ spectra, toxic chemicals may remain unannotated if reference spectra are not available, or if the analytical standard cannot be purchased or synthesized. As a result, only a small fraction of features detected in water samples are confidently annotated or confirmed at the structural level by nontarget workflows (e.g., <1%^14,21^), thereby still limiting the practical use of nontargeted analysis for rapid screening of toxic substances.

Various strategies have been employed to prioritize features of interest in nontargeted data sets, including through study design, such as temporal and spatial trends analysis,^22−26^ case-control comparisons,^27^ or with mass spectral information, such as empirical formula assignment,^28^ mass-defect, or fragmentation flagging.^29^ Since most environmental samples contain thousands of detectable molecular features by nontargeted LC-HRMS, a rapid toxicity-based feature prioritization strategy is desirable. In this direction, Meekel et al.^30^ used structural alerts to trigger high-quality MS^2^ data acquisition with multiple collision energies for presumed toxic compounds based on MS^2^ prescans. Recently, we introduced MS2Tox, a machine-learning tool for prediction of ecotoxicity (i.e., 50% lethal concentration, LC_50_, to aquatic organisms including fish) based only on MS^1^ and MS^2^ spectral data of unknown molecular feature, leveraging on *in silico* tools (i.e., SIRIUS^19^). Molecular fingerprints predicted from mass spectrometric data using SIRIUS software contain structural information. However, toxicity predictions in MS2Tox do not require unequivocal structural annotation or confirmation, and thus MS2Tox enables rapid flagging of the most toxic molecules in complex samples. A similar approach, also using SIRIUS fingerprints,^19^ to prioritize compounds based on their toxicity was presented by Arturi et al.^31^ for toxicological end points in ToxCast/Tox21. Such rapid flagging of potentially toxic substances can be used as a first step to prioritize molecular features for subsequent identification or biological testing,^32^ and could complement traditional approaches such as effect-directed analysis^33,34^ which may be more accurate but are slower, low-throughput, and more resource intensive.

The choice of HRMS data acquisition method is an important consideration for effectively detecting and characterizing chemicals by nontargeted analysis.^35^ For MS^2^ spectral acquisition by LC-HRMS, two general approaches are used: data-dependent acquisition (DDA) and data-independent acquisition (DIA). In DDA, MS^2^ spectra are either acquired based on predefined user criteria or dynamically based on MS^1^ full scan data. One popular approach is suspect screening, whereby MS^2^ spectra are specifically acquired for a defined list of MS^1^ precursor *m*/*z* values selected *a priori*.^36,37^ A dynamic form of DDA acquisition is the TopN scan, whereby the *N* most intense precursor ions (e.g., *N* = 5, 10, or 20) in an MS^1^ scan are selectively fragmented one at a time and the MS^2^ spectrum of each is recorded.^28,38,39^ An advantage of DDA is that it produces relatively clean MS^2^ spectra; however, spectral interferences from cofragmentation of isobaric substances (e.g., *m*/*z* ±0.2 Da^40^) may still occur.^41,42^ Additionally, low-abundance MS^1^ peaks are inherently unlikely to yield corresponding MS^2^ spectra in the TopN approach, particularly in complex samples.^41,43−45^ Due to differences in electrospray ionization efficiency among different chemicals, low peak intensities do not always represent low concentrations in the sample,^46^ and low concentrations of highly toxic compounds may be relevant. Thus, an alternative approach for MS^2^ spectral acquisition is DIA, whereby all precursor ions in a specified mass range(s) are fragmented simultaneously,^47^ and deconvolution software can group MS^2^ fragments together with their MS^1^ precursor ion. DIA data can be acquired by (1) *full-scan DIA*, fragmenting all precursor ions within one MS^2^ scan, (2) *windowed DIA*, fragmenting all precursor ions within specified isolation windows (fixed or variable, narrow or wide, overlapping or not), or (3) various *unconventional DIA methods*.^48^ Although DIA can be more comprehensive for molecular coverage, in practice a limitation may be the utter complexity of the resulting raw MS^2^ data which may not be adequately deconvoluted in data-processing stages.^45,49^

In this work, we compare the performance of DDA (*N* = 10) and DIA (five variable isolation windows) for detection and accurate annotation of a diverse set of 191 reference chemicals spiked to increasingly complex water samples, and examine the subsequent performance of these data sets for MS2Tox LC_50_ prediction in environmental samples. Based on the findings, we furthermore introduce a new parameter in MS2Tox, the CombinedConfidence score, as a relative measure of confidence in LC_50_ predictions for unknown molecular features. Finally, we apply MS2Tox and CombinedConfidence scores to prioritize potentially ecotoxic substances in environmental samples analyzed by both DDA and DIA.

## Materials and Methods

### Water Samples and Analytical Standards

Four water types were spiked with the same mixture of 216 analytical reference chemicals, including e.g. PFASs, surfactants, pharmaceuticals, industrial chemicals, and pesticides, with a wide range of physical properties (e.g., predicted log *P* values from −4.8 to 7.9^50,51^). The detailed list of reference chemicals, sampling locations, and detailed sampling procedures are given in the Supporting Information (SI; Tables S1 and S2). The water types included LC-MS water (Optima grade, Fisher Chemical), groundwater, surface water, and wastewater collected in Sweden. Samples were collected in solvent-cleaned borosilicate flasks in volumes ranging from 40 to 80 mL. The groundwater consisted of pooled grab samples collected in Kalmar, Uppsala, and Västmanlands counties during the spring 2021. Surface water was collected as a grab sample in Lake Mälaren in Stockholm county in autumn 2022. Incoming wastewater was sampled upstream of a major wastewater treatment plant in Stockholm county, representative of household wastewater, and was collected as a weekly composite sample in summer 2021. Transport blank water was 80 mL of the LC-MS water in a precleaned borosilicate flask, taken to the field during sampling and returned and stored with the environmental samples.

### Sample Preparation

Briefly, transport blank and environmental samples were spiked with 28 isotopically labeled internal standards (200 ng/L, Table S2), filtered (0.2 μm, Sartorius Minisart, 15 mm), and transferred to 10 mL glass injection vials. To create spiked environmental samples, a mixture of 216 reference chemicals was added to the samples (100 ng/L each, Table S1), and a corresponding unspiked sample was also kept for each water type. Finally, all samples were spiked with two isotopically labeled injection internal standards (200 ng/L, Table S2). The LC-MS water was prepared in the same way as environmental water types, but without the filtering step. A corresponding instrumental blank sample was prepared with 10 mL of LC-MS water in an injection vial.

### Instrumental Analysis

Water analysis was by online solid-phase extraction (SPE)-LC-HRMS by a modified protocol of Bonnefille et al.^14^ (detailed procedures in the SI). Each sample was injected three times in each ionization mode, in randomized order, except wastewater samples, which were injected at the end of the sequence to avoid any possible cross contamination. Samples were first injected (1 mL) to the online SPE column (Waters Oasis HLB, 15 μm, 2.1 × 20 mm, 80 Å) and subsequently backflushed to a Waters Acquity UPLC BEH C18 analytical column (1.7 μm, 2.1 × 100 mm, 130 Å) with corresponding guard column (1.7 μm, 2.1 × 5 mm, 130 Å). Analytical separation was achieved by gradient elution ultrahigh-pressure liquid chromatography (Ultimate 3000, Thermo Fisher Scientific) at a flow rate of 0.4 mL/min using mobile phases (A) 1 mM ammonium fluoride in water and (B) 100% methanol.

HRMS data were acquired by a Q Exactive Orbitrap HF-X (Thermo Fischer Scientific) operating in electrospray ionization mode. Samples were analyzed in positive and negative ionization modes in separate injections. For the DIA method, acquisition alternated between six scan events, first an MS^1^ full-scan (*m*/*z* 90–1050 Da; 120,000 resolution, fwhm at 200 *m*/*z*), followed by five MS^2^ DIA scans with isolation windows of variable precursor *m*/*z* ranges (30,000 resolution, fwhm at 200 *m*/*z*). Variable sized DIA windows account for uneven precursor ion densities,^52^ and a similar number of windows has shown improved annotation rates in similar water matrices compared to a larger number of DIA acquisition windows.^53^ In DDA, the HRMS alternated between an MS^1^ full-scan (identical to DIA method), and a Top10 MS^2^ program (30,000 resolution, fwhm at 200 *m*/*z*, 10 s dynamic exclusion). A retention time index solution was injected three times per ionization mode for each acquisition method batch.^54^

### Data Processing with MS-DIAL

For peak picking, data alignment, and blank filtering in DIA and DDA, in addition to MS^2^ deconvolution for DIA data, all raw data were processed using MS-DIAL v4.80.^49,55^ Similar parameters were applied for both DDA and DIA (Table S3). Individual sample types (injected in triplicate), along with blank samples (injected in triplicate), were preprocessed in separate batches to minimize false positives in gap-filling steps. To minimize false positives, only features detected in each triplicate injection per sample type were kept in subsequent data analysis. For further data processing, only those ions assigned as molecular ions ([M + H]^+^, [M – H]^−^) or sodium adducts ([M + Na]^+^) in MS-DIAL were used for comparison of the methods. Retention times of the spiked reference standards were recorded by manual inspection of spiked LC-MS grade water chromatograms. Features representing the spiked chemicals were extracted from MS-DIAL output using R (v4.2.2) based on criteria of retention time (±30 s) and MS^1^ precursor *m*/*z* error (<5 ppm).

### Data Processing with SIRIUS+CSI:FingerID and R

To evaluate MS^2^ spectral quality, MS^2^ data for spiked reference standards were structurally annotated using SIRIUS+CSI:FingerID (v5.6.2 and v5.6.3).^19,56^ This software was also used to output molecular fingerprints from the MS^2^ spectra for LC_50_ predictions. LC_50_ values were predicted using the function *FishLC50Prediction(LC50mode=”static”)* in R-package MS2Tox version 0.3.2.^32^ With this function, LC_50_ values are predicted in log-mM units and were converted to mg/L using the molecular mass of the predicted formula. A new confidence score, CombinedConfidence, was calculated by averaging scaled values (range 1–5) of absoluteMassErrorPrecursor, TopCSIDcore, and ConfidenceScore from SIRIUS+CSI:FingerID; this was used to describe the confidence of predicted LC_50_s. Predicted molecular formulas without the scores (e.g. ConfidenceScore is assigned only to first rank structure in SIRIUS+CSI:FingerID) were assigned a score equal to the lowest score in this data set.

### Annotation of Prioritized Features

For the case study, high-confidence potentially toxic molecular features in the spiked environmental samples having a MS2Tox predicted LC_50_ < −2 log-mM, and a CombinedConfidence score >3, were prioritized for annotation. As a comparison, high-confidence features predicted to have low toxicity were also selected using criteria of MS2Tox predicted LC_50_ > 0 log-mM and CombinedConfidence >3. After prioritization, the features were manually investigated, and integrated noise (i.e., false peaks) or impurities of spiked chemicals were removed from the prioritization list by comparison to corresponding unspiked samples of the same water type. MS2Tox predictions for target analytes (*N* = 216; Table S1) were additionally performed in the unspiked samples for the case study, following the same procedure as described for the spiked samples. For feature annotations at confidence level 2a, according to confidence levels of Schymanski et al.,^57^ spectral library matching to MassBank Europe^18,58^ was performed in MS-DIAL (v. 4.80, Mass tolerance 0.002 Da in MS^1^ and 0.005 in MS^2^, 60% identification score cutoff). All probable structures (level 2a) were further evaluated for supporting evidence using the retention time index, and only structures falling within box1 (”candidate accepted”)^54^ were considered for the case study. For evaluating MS2Tox predicted toxicities for level 2a annotated structures, 96 h LC_50_ values of fathead minnow were predicted from the structure with the CompTox Chemicals Dashboard prediction tool (v2.2.1).

### Data and Code Availability

R-code for MS2Tox, using command line for SIRIUS+CSI:FingerID calculations, and ConfidenceScore are available in MS2Tox GitHub (https://github.com/kruvelab/MS2Tox). Tables with predicted toxicities and formulas for acquired features are presented in the SI.

## Results and Discussion

### MS^1^ and MS^2^ Feature Detection Rates in DDA and DIA

Detection rates by DDA and DIA MS^2^ acquisition methods were evaluated based on detection of spiked chemicals in MS^1^ and MS^2^, as well as the total number of nontargeted features detected in water types of increasing complexity. Of the 216 spiked chemicals, 191 were recovered by the online SPE, and thus only this subset of reference chemicals was considered in the comparison of DDA and DIA (Table S1). After preprocessing, data filtering, and blank filtering in MS-DIAL, 161 MS^1^ precursor ions out of the subset of 191 reference chemicals were detected at the correct retention times by both DDA and DIA methods for the clean LC-MS water matrix. Thus, some analytes were lost in the data processing steps, such as by removal through blank filtering or incorrect adduct assignment by MS-DIAL. For example, in positive ionization mode the [M + H]^+^ precursor ion of memantine was detected but was assigned as the [M + NH_4_]^+^ adduct in MS-DIAL, in both DIA and DDA acquisition methods.

**Figure 1:**
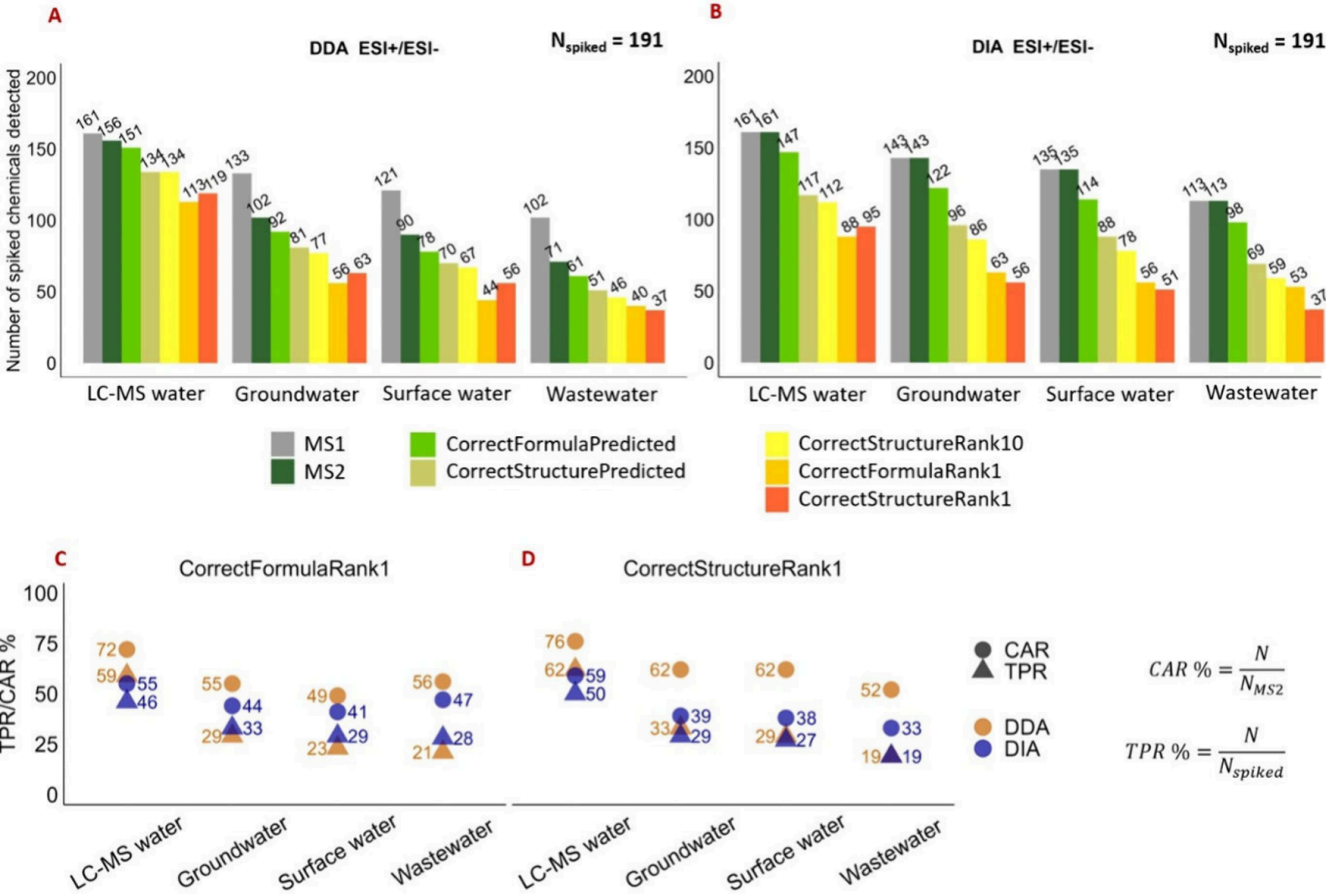
(A,B) Number of spiked reference chemicals detected in full scan MS^1^ and with acquired MS^2^ spectrum, separated by MS^2^ acquisition method and water sample type. The corresponding numbers of correct *in silico* formulas and structural annotations are shown as CorrectFormulaPredicted (correct formula is predicted by SIRIUS), CorrectStructurePredicted (correct structure is predicted with SIRIUS), CorrectFormulaRank10 (correct structure is ranked 1–10), CorrectFormulaRank1 (correct formula is ranked highest), and CorrectStructureRank1 (correct structure is ranked highest). (C,D) True positive rate (TRP) and correct annotation rate (CAR) for rank1 formula and structure.

In spiked LC-MS water, groundwater, surface water, and wastewater we detected 1889, 5453, 5709, and 19166 MS^1^ features, respectively, with the DIA method (both ionization modes). With DDA, we detected 2349, 4681, 5129, and 14530 MS^1^ features, respectively. Considering spiked chemicals, fewer reference chemicals were detected in MS^1^ with increased sample complexity by both DDA and DIA (i.e., clean water > groundwater > surface water > wastewater), but the diminished performance was more pronounced for DDA, and DIA resulted in more detections in MS^1^ in the environmental samples (Figure 1). Considering all nontarget features and spiked reference chemicals together, overall a lower number of MS^1^ features was detected in environmental samples by DDA compared to DIA (75.6% to 90.0% of total feature count in DIA; Table S4). One potential explanation for this was the inherently longer scan cycle time of DDA (550 ms) than DIA (300 ms); however, the number of scans per peak for spiked chemicals were satisfactory for both MS^2^ acquisition methods in all water matrices, generally exceeding 12 scans per peak for a 12 s wide peak.

The number of molecular features with acquired MS^2^ spectra was higher for DIA than for DDA. This is consistent with Zha et al.,^59^ who reported that 80.8% of metabolites in biological samples had corresponding MS^2^ spectra in DIA, compared to only 54.6% in DDA. We observed an even greater difference, where 99–100% of nontargeted features yielded a corresponding MS^2^ spectrum in DIA and only 24–50% in DDA (Table S4). Results depended on water type and ionization mode, i.e. in spiked wastewater we detected 19,067 features with acquired MS^2^ spectra (positive and negative mode combined) in DIA, while DDA yielded only 6227 features. For DDA, no association between number of MS^1^ features detected and number of MS^2^ spectra triggered was observed; however, the number of spiked chemicals with an acquired MS^2^ spectrum decreased with increasing complexity (Figure 1A). A similar trend was observed for spiked chemicals by the DIA method, but to a smaller extent (Figure 1B).

Considering the acquisition of MS^2^ spectra for the 191 spiked reference chemicals at 100 ng/L, only minor differences were observed by the acquisition method for the LC-MS water, wherein 156 reference chemicals had corresponding MS^2^ spectra in DDA (81.7% coverage), compared to 161 for DIA (84.3% coverage). In environmental waters, MS^2^ spectra were acquired for only 71–102 of 191 reference chemicals (37.2–53.4% coverage) by DDA (Figure 1A), compared to 113–143 chemicals (59.2–74.9%) by DIA (Figure 1B), depending on sample complexity. In comparison to detected features in MS^1^, 100% of the MS^1^ detected reference compounds had a corresponding MS^2^ spectrum in DIA for all environmental water sample types, but for DDA only 70–77% had a corresponding MS^2^ spectrum (Figure 1A). In the cleanest water matrix studied, the LC-MS water, we observed that for the reference chemicals with an MS^2^ spectrum acquired only by DIA (*N* = 10), these had two orders of magnitude lower median peak area (4.5 × 10^6^) compared to reference chemicals with an MS^2^ spectrum acquired by both approaches (*N* = 151; 3.1 × 10^8^) or only DDA (*N* = 5; 6.7 × 10^8^). In surface water, however, the observed median peak areas were comparable. On the other hand, we observed a shorter median retention time for the chemicals having an MS^2^ spectrum that was only acquired by DIA (*N* = 47; 7.5 min) compared to those with MS^2^ acquired by both methods (*N* = 88; 10.9 min).

Considering MS^1^ and MS^2^ data overall, our results confirm that DIA gave higher detection rates than DDA for nontargeted acquisition in complex environmental samples. Although direct comparison with previously published nontargeted water analysis is impossible, similar trends have been observed in the metabolomes and exposomes. Barbier Saint Hilaire et al.^60^ reported a similar trend comparing DDA and DIA, whereby DIA resulted in fragmentation spectra of all 72 targeted low-mass metabolites in human serum, but only 61% of these had fragmentation spectra by DDA. Additionally, Wu et al.^61^ could identify 66 of 68 veterinary drug residues in aquacultured eel with more than one product ion with DIA, compared to only 48 with target DDA, due to the absence of recorded MS^2^ spectra.

### Performance of DDA and DIA for Annotation of Structure and Formula

To compare the performance of DDA and DIA in formula and structural annotations, we evaluated the true positive rates (TPRs) of the first ranked formula as well as structural annotations using SIRIUS+CSI:FingerID and spectral library matching to MassBank Europe. The TPRs were calculated as the proportion of the 191 spiked reference chemicals with a correct first-ranked formula and first-ranked structure annotated.

Considering first-ranked formula assignments (Figure 1, orange bars named CorrectFormulaRank1), DDA and DIA resulted in TPRs of 59.2% and 46.1% in LC-MS water, respectively, suggesting a higher quality of MS^2^ spectra by DDA. However, higher TPRs were evident for DIA than for DDA in all environmental waters. Specifically, TPRs in DIA were 33.0%, 29.3%, and 27.7% in groundwater, surface water, and wastewater, respectively, compared to 29.3%, 23.0%, and 20.9% in DDA (Figure 1C). Additionally, we observed lower TPRs among halogenated chemicals in formula predictions with SIRIUS. Here, 20 out of 36 reference chemicals that never yielded correct first-ranked formula predictions in both DDA and DIA were halogenated, including 12 that contained fluorine, such as the PFASs (*N* = 9) and fluorinated pharmaceuticals (*N* = 3).

For accurate *in silico* structural annotations with SIRIUS+CSI:FingerID, DDA generally had higher TPRs than DIA despite the fact that DDA yielded lower numbers of detected reference chemicals with MS^2^, suggesting increased MS^2^ spectral quality by DDA (Figure 1D). More specifically, with DDA data from analysis of LC-MS water, groundwater, surface water, and wastewater, the correct first-ranked structures were annotated for 119 (TPR 62.3%), 63 (33.0%), 56 (29.3%), and 37 (19.4%) reference chemicals, respectively. Correspondingly in DIA, correct first-ranked structures were annotated for 95 (TPR 49.7%), 56 (29.3%), 51 (26.7%), and 37 (19.4%) with the DIA data (Figure 1D). Nijssen et al.^62^ obtained similar results, attributed to higher MS^2^ spectra quality for DDA data, when comparing true positive annotations of 32 veterinary drugs and pesticides spiked in solvent and animal feed extract testing four different annotation tools (one mass spectral library, three *in silico* tools). Nevertheless, in wastewater, the most complex water samples analyzed here, the TPR was the same (19.4%) for DDA and DIA, owing to the trade-offs of method detection sensitivity and MS^2^ spectral quality by the two protocols. Comparison of structural annotation accuracy by SIRIUS for positive and negative ionization modes reveal only minor differences for LC-MS water, independent of the MS^2^ acquisition mode. However, a tendency of lower TPRs were observed for the spiked chemicals in the environmental samples in the negative ionization mode, compared to the positive ionization mode. The divergence is especially noticeable for the DDA method. For example, in surface water, an MS^2^ spectrum was acquired for 56 compounds in positive mode and 51 in negative mode, while correct rank 1 structure annotations were observed for 42 and 21 compounds, respectively.

Regarding the performance of MS^2^ spectral library matching, higher TPRs were observed for DIA in all water sample types, with decreasing TPRs with increasing sample complexities. In LC-MS water, groundwater, surface water, and wastewater, 138 (TPR 72.3%), 88 (46.1%), 81 (42.2%), and 65 (34.0%) spiked reference chemicals were correctly matched to MassBank Europe by DDA, respectively (Table S5). In DIA, the corresponding numbers were 139 (TPR 72.8%), 98 (51.3%), 96 (50.3%), and 77 (40.3%). Higher TPRs in *in silico* formula assignments and empirical spectral library matching by DIA in environmental matrices suggest that these approaches are less sensitive to MS^2^ spectral interference than *in silico* structural assignment, and that overall DIA performance benefited from the higher detection rates of the spiked reference chemicals. However, spectral libraries are biased toward well-known and frequently monitored chemicals, such as the spiked reference chemicals, and contain many fewer candidates for matching compared to the more comprehensive *in silico* structure libraries, potentially explaining the differences in TPRs by these two complementary approaches.

### Assessment of DDA and DIA MS^2^ Spectral Quality

For evaluation of MS^2^ spectral quality, we assessed *correct annotation rate* (CAR%), comparing the ratio between correct rank1 structural annotations and the number of spiked chemicals with an acquired MS^2^ spectrum in the samples (Figure 1, dark green bars named MS^2^), rather than TPR, which considered all 191 detectable reference chemicals. The DDA method had higher CARs for *in silico* structural annotation in SIRIUS, ranging from 52.1% to 76.3%, compared to 32.7% to 59.0% with DIA (Figure 1D). Additionally, in spectral library matching the DDA method yielded higher CARs than DIA: 88.5%, 86.3%, 90.0%, and 91.5% for LC-MS water, groundwater, surface water, and wastewater, respectively, compared to 86.3%, 68.5%, 71.1% and 68.1%. These data suggest a higher spectral quality for MS^2^ spectra acquired by DDA, and the same has been suggested earlier by Barbier Saint Hilaire et al.^60^ based on higher dot products for DDA spectra matched with in-house reference spectra compared to DIA spectra. However, it should be kept in mind, as discussed above, higher TPRs were observed for the DIA method due to the superior detection rates of MS^2^ features, suggesting a balanced performance in MS^2^ acquisition and spectral quality also by DIA.

### Comparative Performance of MS2Tox with DDA and DIA

MS2Tox predicts fish LC_50_ for detected features based on MS^1^ and MS^2^ spectral data, independent of structural identification. Nevertheless, MS2Tox depends on input of accurate molecular fingerprints predicted from formula and MS^2^ spectra with SIRIUS+CSI:FingerID. Thus, here we compared predicted LC_50_ values from MS2Tox based on MS^2^ data acquired here by DIA and DDA to the LC_50_ values predicted by MS2Tox based on the correct structures. Note that the predicted LC_50_ values were not compared to experimental LC_50_ values because only 35 of 191 spiked reference chemicals had experimental LC_50_ values in CompTox^63^ databases. The accuracy of MS2Tox has been previously shown by Peets et al.^32^ and thus the usage of predicted toxicity values from known structures allowed us to utilize other reference compounds besides the 35 in the comparative analysis of DDA and DIA. Figures comparing predicted toxicity values and experimental values are shown in SI Figures S2 and S3.

**Figure 2:**
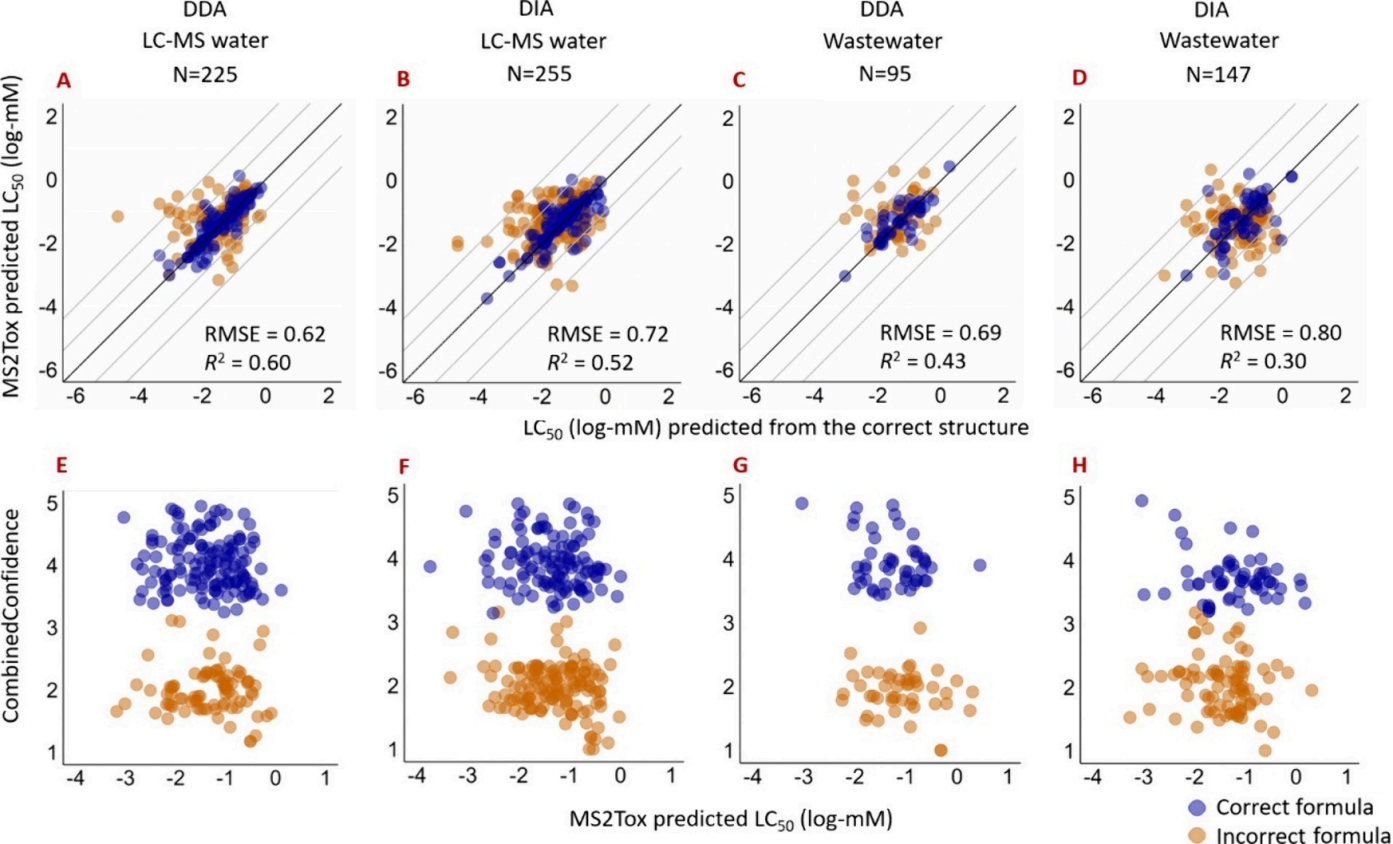
(A–D) Comparison of MS2Tox predicted LC_50_ values from MS^2^ data in DDA or DIA in LC-MS water or wastewater, versus MS2Tox predicted LC_50_ values from correct structures. Each point in all plots (*N*) is an LC-HRMS feature corresponding to the spiked reference chemical; blue points correspond to MS^2^ features with correct molecular formula prediction, while orange points correspond to incorrect molecular formula prediction. (E–H) Predicted LC_50_ values with assigned CombinedConfidence from precursor mass error, top CSI score, and Confidence score from SIRIUS+CSI:FingerID output.

Due to the higher MS^2^ detection rates with DIA, LC_50_ values were predicted by MS2Tox for more LC-HRMS features with DIA, compared to DDA (Figure 2); note that the number of extracted features was higher than the number of detectable spiked reference standards, due to some chemicals being detected in both positive and negative modes as well as detection as adducts. The LC_50_ values predicted from DDA spectra had somewhat lower RMSE (0.62 to 0.76 log-mM depending on the matrix) compared to DIA (0.71–0.80 log-mM), suggesting higher fingerprint accuracy in DDA. Manually checking spiked reference chemicals detected in groundwater in positive ionization mode showed that precursor ion peak areas were lower for those only detected by DIA (*N* = 17, median 1.38 × 10^7^) compared to those detected only by the DDA method (*N* = 7, median 1.38 × 10^9^). Lower precursor ion intensities could lead to poorer MS^2^ quality^47^ and might explain the somewhat higher RMSE by DIA. RMSE values for LC_50_ predicted from MS^2^ spectra were below 0.97 log-mM for both correctly and incorrectly predicted formulas with both acquisition methods (Table 1). However, we observed a lower correlation between LC_50_ values predicted based on MS^2^ and structure when incorrect formula was assigned, compared to when the correct formula was assigned (Table 1). This was expected because molecular formula annotation affects the fragmentation tree, and thus the molecular fingerprint predicted with SIRIUS+CSI:FingerID. The issue of significant errors in toxicity predictions, despite correct chemical formula annotations, can be attributed to the incomplete MS^2^ (tandem mass spectrometry) data, which can lead to inaccurate fragmentation trees and, consequently, molecular fingerprints. For instance, one of the highest errors observed was for the spiked chemical 2,6-di-*tert*-butyl-4-(hydroxymethyl)phenol in DIA mode. While SIRIUS correctly assigned the chemical formula C_15_H_24_O_2_, the cosine similarity between SIRIUS predicted fingerprint and correct structure fingerprint was only 0.33, indicating that MS^2^ data were insufficient for correct fingerprint prediction. This is also reflected in the fact that the top1 candidate structure was 2,2-bis(4-oxocyclohexyl)propane. Despite sharing the same chemical formula, these two compounds have vastly different structures. The spiked chemical possesses an aromatic ring with hydroxy groups, whereas the predicted structure features nonaromatic rings and ketone groups. This example highlights how incorrect toxicity predictions can arise even when the chemical formula is correctly assigned. The primary issue is the accuracy of the predicted molecular fingerprints, possibly due to incomplete fragmentation data.

**Table 1:** Statistical Comparison of the Toxicity Predictions from MS^2^ Spectra and Structure with MS2Tox in Different Sample Matrices^a^

Acquisition	Sample matrix	RMSE all	*R*^2^ for all	RMSE correct formula	*R*^2^ for correct formula	RMSE incorrect formula	*R*^2^ for incorrect formula
DDA	LC-MS water	0.62	0.60	0.28	0.90	0.95	0.26
DDA	Groundwater	0.68	0.49	0.36	0.86	0.89	0.08
DDA	Surface water	0.76	0.39	0.35	0.88	0.97	–0.06
DDA	Wastewater	0.69	0.43	0.35	0.84	0.92	0.12
DIA	LC-MS water	0.72	0.52	0.41	0.81	0.90	0.34
DIA	Groundwater	0.80	0.42	0.52	0.70	0.93	0.29
DIA	Surface water	0.80	0.39	0.46	0.81	0.92	0.14
DIA	Wastewater	0.80	0.30	0.56	0.65	0.93	0.02

a RMSE and *R*^2^ are calculated when comparing MS2Tox values calculated from SIRIUS fingerprints to MS2Tox calculated using the correct structure. A lower RMSE and higher *R*^2^ show higher agreement.

### CombinedConfidence Score: Evaluation of MS2Tox Prediction Reliability

For the discussion above, the true formula and structure of the spiked chemicals were always known; however, in real-world analysis of environmental samples it would be useful to accompany the MS2Tox LC_50_ predictions with a relative measure of confidence. SIRIUS+CSI:FingerID reports several scores or values that can be useful in this regard: for example, the mass error between theoretical mass of predicted formula and *m*/*z* of detected peak, as well as a SiriusScore, which is used to rank the predicted formulas. In this work, mass accuracy of precursor *m*/*z*, ConfidenceScore, and topCSIscore were evaluated and combined into a combined confidence score (CombinedConfidence) to accompany predicted LC_50_ values from MS2Tox. CombinedConfidence scores were observed to be generally higher for correctly assigned formulas than for incorrectly assigned formulas (Figure 2E–H), and CombinedConfidence yielded a clear separation of correct and incorrect formula predictions.

CombinedConfidence scores (Figure 2E–H) can also be used to evaluate reliability of the LC_50_ predictions for unknown features. Molecular features with high CombinedConfidence, and higher toxicity (upper left corner in each panel of Figure 2E–H) can be prioritized for structural annotation with higher reliability. In contrast, the upper right corners of these plots contain features considered to have low toxicity, with high confidence, and thus are less likely to contribute to toxicity of the water samples. Features with low CombinedConfidence scores should be treated with caution and cannot necessarily be ruled out as high or low toxicity features.

### Prioritization of Toxic Compounds in Environmental Water Samples

**Figure 3:**
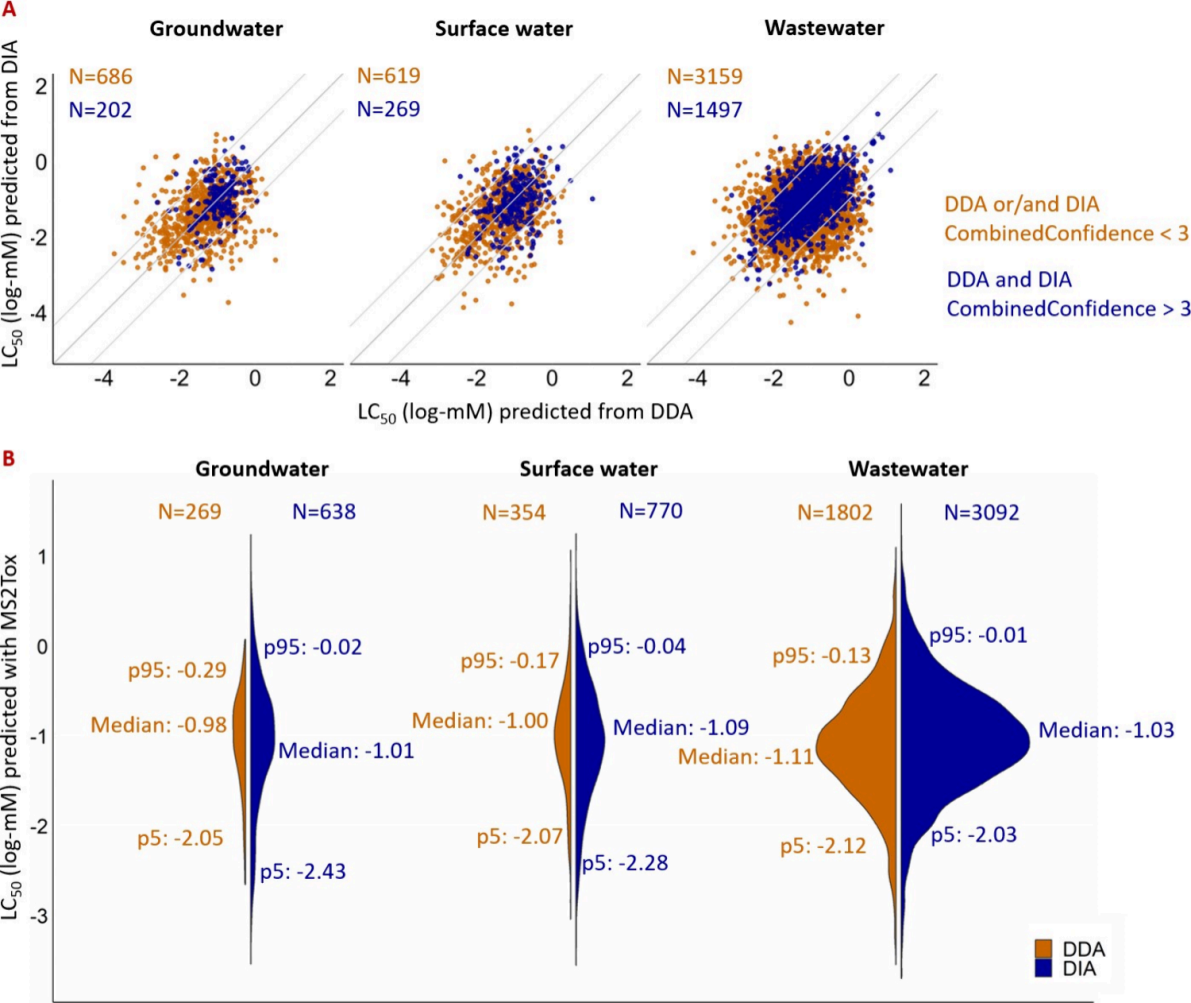
Toxicity and CombinedConfidence score prediction for detected nontargeted features from three environmental samples. (A) Comparison of predicted toxicity values for overlapping features from DDA and DIA measurements. Overlap was considered for features with a retention time difference below 1 min and precursor *m*/*z* difference below 5 ppm; therefore, features from one acquisition data set can match multiple features from another. Features are divided into two groups based on assigned CombinedConfidence score for the toxicity prediction. (B) Distribution of predicted LC_50_ values for detected nontargeted features (*N*) with CombinedConfidence >3. The 5th centiles (p5), median, and 95th centiles (p95) of LC_50_ are labeled.

As a proof of concept, MS2Tox was applied to prioritize nontargeted features in the spiked environmental samples based on the predicted fish LC_50_. LC_50_ values were predicted with MS2Tox for all features assigned by MS-DIAL as [M + H]^+^, [M + Na]^+^, or [M – H]^−^ that had SIRIUS predicted fingerprint from MS^2^ data. Altogether 936, 1034, and 4486 LC_50_ values were predicted from DDA data, whereas 3605, 3650, and 13094 LC_50_ values were predicted from DIA data for groundwater, surface water, and wastewater, respectively. Out of these, 791, 795, and 4185 features from DIA overlapped with 724, 740, and 3807 features from DDA, with a retention time difference of <0.5 min and precursor *m*/*z* difference of <5 ppm and matched assigned adduct. The nonmatching numbers across the two acquisition methods here are explained by multiple matches per feature with the automatic workflow and set criteria. All overlapping features are compared based on their predicted LC_50_ values. Features with CombinedConfidence scores exceeding 3 generally had better agreement in toxicity values between the two acquisition modes (Figure 3A), suggesting higher agreement of molecular fingerprint predicted with SIRIUS for higher confidence features.

Considering only those detected features with high CombinedConfidence (>3) in groundwater, surface water, and wastewater, we examined the predicted toxicities of 269, 354, and 1802 features from DDA data sets and 638, 770, and 3092 from DIA, respectively. The median predicted LC_50_ values across sample types were similar; from −1.12 to −0.98 log-mM (i.e., 23 to 31 mg/L for molar mass of 300 Da) in both MS^2^ acquisition methods (Figure 3B). For prioritization we set the LC_50_ threshold for *high toxicity features* to <−2 log-mM, and as a comparison in this proof of concept we defined *low toxicity features* as those with LC_50_ > 0 log-mM.

Prioritizing features with high toxicity (<−1 log-mM and CombinedConfidence >3), after manual exclusion of integrated noise or impurities associated with spiked chemicals, the final data set consisted of 10, 16, and 117 nontargeted features in the groundwater, surface water, and wastewater samples based on DDA MS^2^. In the same samples analyzed by DIA, 41, 32, and 144 features were prioritized, accordingly. The overlap of high toxicity features prioritized by both DIA and DDA was low overall: none of the prioritized features by DDA were prioritized by DIA in the groundwater, 12.5% for surface water, and 17.9% for wastewater. Additionally, to reduce the chance of overlooking environmentally relevant spiked chemicals, we performed target screening of the 191 reference chemicals in the corresponding nonspiked environmental samples. Here, no target analytes were prioritized in either the DDA or DIA data set, but seven reference chemicals were detected and prioritized as high toxicity by MS2Tox (predicted LC_50_ < −2 log-mM) with one of the acquisition methods (Table 2). For target analytes, the calculated CombinedConfidence for correct formula predictions were >3 and for incorrect formula predictions were <3.

Altogether, 42 prioritized high toxicity features had spectral matches to MassBank Europe, whereas 13 of these are included in further discussion since only these were supported by retention time index as accepted candidates (level 2a; Table 2). For target analytes, the calculated CombinedConfidence for correct formula predictions were >3 and for incorrect formula predictions were <3. Out of these 13 probable structures (level 2a), only the natural product diosgenin overlapped between DDA and DIA. Diosgenin naturally occurs in multiple plant species, is used as a precursor for the synthesis of steroids, and has multiple pharmaceutical activities.^64^ MS2Tox predicted fish LC_50_s of 0.93 and 3.6 mg/L for diosgenin (2a) from the DDA and DIA spectra, respectively, and based on structure a LC_50_ of 0.1 mg/L was predicted (Table 2). Despite the difference of 1 order of magnitude in LC_50_ predicted from MS^2^ and structure, MS2Tox prioritized diosgenin as a potentially toxic chemical in the samples.

**Table 2:**
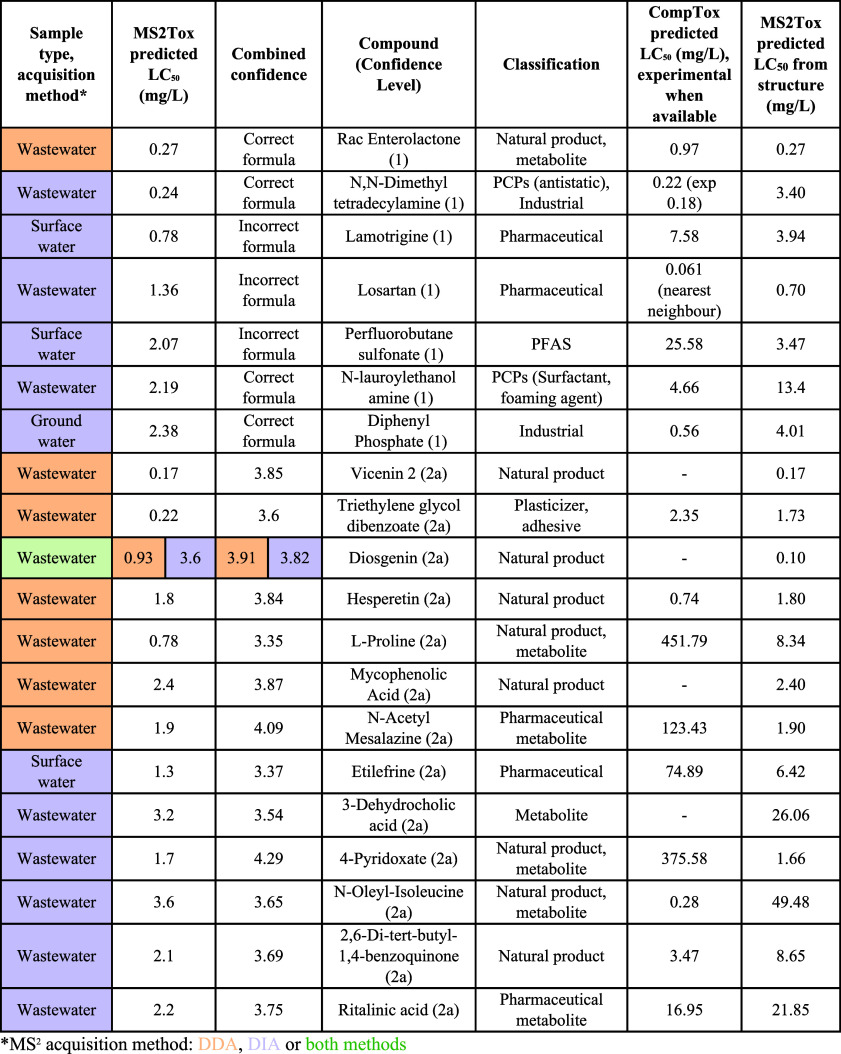
Annotated Structures (Reference Chemicals and Level 2a^57^) Prioritized with MS2Tox (High Toxicity + High CombinedConfidence), Predicted Fish LC_50_ with Accompanying CombinedConfidence for Nontarget Features (2a) or Formula Prediction Information for Reference Chemicals (1), Classification, and 96 h Fathead Minnow LC_50_ predicted by CompTox Chemicals Dashboard, and by MS2Tox from Annotated Structure^a^

a InChlKeys are provided in Table S6.

To assess the performance of the MS2Tox fish LC_50_ predictions among detected features, we applied CompTox to predict the fish LC_50_ values from the structure as comparison for the probable structures (*N* = 13, level 2a) and detected reference chemicals (*N* = 7, Table 2). Out of these, vicenin 2 (level 2a), annotated in wastewater by DDA, was assigned the most toxic fish LC_50_ (0.17 mg/L) by MS2Tox. Vicenin 2 is a natural product in plants and has also shown to possess a variety of pharmacological activities.^65−68^ No LC_50_ values are reported or could be predicted in CompTox, but the corresponding MS2Tox predicted toxicity based on the structure of vicenin 2 was identical (0.17 mg/L) to prediction from the DDA spectrum. Three reference chemicals had predicted LC_50_ values below 1 mg/L (Table 2). Out of these, enterolactone (reference chemical acquired in DDA) and *N*,*N*-dimethyltetradecylamine (reference chemical acquired in DIA) yielded fish LC_50_ values comparable to the values predicted by CompTox (Table 2). Lamotrigine (reference chemical; DIA), on the other hand, showed an order of magnitude disagreement (0.78 mg/L by MS2Tox; 7.58 with CompTox), which is likely explained by an incorrect formula assignment in SIRIUS. This is supported by the fact that the LC_50_ predicted by MS2Tox from the correct structure was 4.06 mg/L. We also observe a significant disagreement for predicted fish LC_50_ for perfluorobutanesulfonate (reference chemical, MS^2^ acquired in DIA), which also was assigned the incorrect formula by SIRIUS (Table 2). Overall, there was a statistically significant difference between MS2Tox and CompTox predicted fish LC_50_ values (Wilcoxon signed rank test, *p* < 0.05) due to high differences for one outlying annotation, l-proline (Table 2). Excluding l-proline from the comparison, no significant difference between MS2Tox and CompTox predicted fish LC_50_ values were observed (Wilcoxon signed rank test, *p* > 0.05). For l-proline (level 2a; DDA), MS2Tox predicted a low LC_50_ of 0.78 mg/L, whereas CompTox prediction based on structure was 452 mg/L (Table 2). We detected l-proline only in negative ionization mode, with only two matched fragment ions to the reference spectrum. However, l-proline has been reported to ionize more efficiently in positive ionization mode,^69,70^ and therefore an assumption can be made that the structural annotation was incorrect leading to a biased CompTox prediction, rather than a poor toxicity prediction. For other chemicals with divergent predicted toxicity values, no underlying structural or functional similarities were identified that could account for the observed differences.

For a comparison, we also investigated low toxicity nontargeted features (predicted LC_50_ > 0 log-mM, CombinedConfidence >3; *N* = 87 in DDA; *N* = 243 in DIA) and reference chemical targets (predicted LC_50_ > 0 log-mM, *N* = 1 in DIA), and as above only reference chemicals or 2a annotations (with supporting evidence using retention time index) were considered (Table S3; *N* = 13). Favorably, we did not annotate any high toxicity features within the subset defined as low toxicity features, which in real applications would not be prioritized as hazardous with MS2Tox. Comparing the toxicity of high and low toxicity features ( and Table S7), the two groups are significantly different based on the MS2Tox predictions (*p* < 0.05). Comparing predicted LC_50_ values for the same features using CompTox, significant difference is observed only if l-proline (2a) and 4-pyridoxate (2a) are excluded as outliers (*p* < 0.05). Due to the low number of reference chemicals and 2a annotated structures with retention time index confirmations, no difference between DDA and DIA acquisition methods can be observed.

### Implications of Our Findings

To conclude, DDA MS^2^ spectra generally had higher correct annotation rates (i.e., ratio between correct rank1 annotations and number of reference chemicals with acquired MS^2^ spectra) in structural annotations using SIRIUS:CSI:FingerID and spectral library matching, suggesting better MS^2^ spectral quality compared to DIA. Additionally, lower RMSE for LC_50_ values predicted from DDA spectra suggest higher fingerprint accuracy from DDA spectra, compared to DIA spectra. Although MS^2^ spectral quality was shown to be an important factor for the accuracy of toxicity predictions, DIA advantages cannot be ignored, as DIA yields higher MS^2^ detection rates for targeted and nontargeted features. In combination with satisfactory MS^2^ spectral quality, this led to a higher true positive rate in DIA (i.e., percentage of correct rank1 annotations among 191 reference chemicals) of formula predictions and spectral library matches than in DDA. MS2Tox can successfully be applied to prioritize potentially toxic molecules in environmental samples; however, it should be applied with understanding of limitations as with other *in silico* prediction tools. Nevertheless, MS2Tox relies on correct first-ranked formula predictions from SIRIUS, affecting the accuracy of LC_50_ predictions. To mitigate this limitation, the new CombinedConfidence score gives the user an indication of the fingerprint accuracy. In terms of MS2Tox, the formula derived from the first-rank structure could be utilized instead of the first-rank formula. Future research could focus on predicting toxicity directly from MS^2^ data, eliminating the need for SIRIUS software.

Separate data preprocessing of individual samples poses a limitation to fully benefiting from the nontargeted data processing workflows. For example, processing more samples together would likely enhance MS^2^ spectral quality because the sample having the highest peak intensity (and thus higher spectral quality) would be used for the MS^2^ deconvolution. Similarly, data alignment and gap filling among multiple samples could increase the number of features with an acquired MS^2^, and thereby broaden the range of considered chemicals. Nevertheless, here separate data processing of samples was necessary to compare the MS^2^ acquisition performances in water samples of variable complexities. For future applications of nontarget workflows with MS2Tox feature prioritization, iterative DDA workflows can potentially be applied for comprehensive high-quality MS^2^ data acquisition for a small number of samples or pooled samples. However, due to long analysis time with repeated measurements per sample, we argue that it is beneficial to apply DIA workflows followed by spectral deconvolution for a nonbiased comprehensive MS^2^ acquisition for a large number of samples. It is therefore essential to optimize DIA and DDA workflows to ensure higher MS^2^ quality, both *in operando* (i.e., instrumental parameters) and *in silico* (i.e., spectral deconvolution), to ensure higher accuracies in both structural annotations and toxicity predictions.

## Supporting Information Available

Additional materials and methods, additional results, standard chemical data, and data analysis parameters (PDF)
Results data for creating the main figures (ZIP)

## Abbreviations

CAR: correct annotation rate
DDA: data-dependent acquisition
DIA: data-independent acquisition
LC-HRMS: liquid chromatography high-resolution mass spectrometry
LC_50_: lethal concentration to 50% of test organisms
PCPs: personal care products
PFAS: per- and polyfluoroalkyl substances
RMSE: root mean squared error
SPE: solid-phase extraction
TPR: true positive rate
