# Supplementary material for: Evaluation of Nontargeted Mass Spectral Data Acquisition Strategies for Water Analysis and Toxicity-Based Feature Prioritization by MS2Tox
Source: Environ Sci Technol. 2024 Sep 19;58(39):17406–18. doi: 10.1021/acs.est.4c02833 (PMC11447898; doi:10.1021/acs.est.4c02833)
Supplement: Supplementary file 1 — es4c02833_si_001.pdf [file es4c02833_si_001.pdf]

Supplementary information for article

"Evaluation of non-targeted mass spectral data acquisition strategies for water analysis and toxicity-based feature prioritization by MS2Tox"

Pilleriin Peets[1,3†], May Britt Rian[2†], Jonathan W. Martin[2,4], Anneli Kruve[1,2*]

[1] Department of Materials and Environmental Chemistry, Stockholm University, Svante Arrhenius Väg 16, SE-106 91, Stockholm, Sweden

[2] Department of Environmental Science, Stockholm University, Svante Arrhenius Väg 16, SE-106 91 Stockholm, Sweden

[3] Institute of Biodiversity, Faculty of Biological Science, Cluster of Excellence Balance of the Microverse, Friedrich-Schiller-University Jena, 07743, Jena, Germany

[4] National Facility for Exposomics, Metabolomics Platform, Science for Life Laboratory, Stockholm University, Solna 171 65, Sweden

* Corresponding author anneli.kruve@su.se

† These authors contributed equally to this paper.

Summary: 29 pages, 5 figures, 7 tables and 3 captions for supplementary datasheets.

SI materials and methods
Materials

LC-MS optima grade methanol (Fisher Scientific), LC-MS optima grade water (Fisher Scientific) and ammonium fluoride (Honeywell Fluka) was applied as LC mobile phases. Analytical standards (N=216, table S1) were chosen based on previously reported occurrence in relevant water matrices [1-8], concerns as water pollutants [9-12] and availability from earlier projects [13-15]. Internal standards (N=30, table S1) was chosen based on analytical coverage, derived from Bonnefille at al. [13]. Out of 216 spiked chemicals, 191 were recovered with LC-HRMS analysis. The majority (17 out of 25) of the non-recovered chemicals with the applied on-line SPE method were anionic in the applied conditions, and are therefore potentially outside the chemical-space covered by the HLB based extraction. Furthermore, five dyes remained undetected at 100 ng/L due to method detection limits exceeding 100 ng/L. Additionally, some chemicals were excluded due to high background levels (e.g. monoisobutyl phthalate and sodium lauryl sulfate).

Table S1. Analytical standards (N=216), CAS number, supplier. Out of these, 191 were recovered at 200 ng/L with the applied method. Standards not recovered with applied method are marked with grey.

Analytical standard	CAS number	Supplier
Aniline	62-53-3	Sigma-Aldrich
ε-Caprolactam	105-60-2	Sigma-Aldrich
1H-Benzotriazole	95-14-7	Sigma-Aldrich
4-Methyl-1H-benzotriazole	29878-31-7	Sigma-Aldrich
5-Methyl-1H-benzotriazole	136-85-6	Sigma-Aldrich
Glyphosate	1071-83-6	Sigma-Aldrich
4-Toluenesulfonamide	70-55-3	Sigma-Aldrich
Gabapentin	60142-96-3	Sigma-Aldrich
p-Toluenesulfonic acid	6192-52-5	Sigma-Aldrich
2,4-Dinitrophenol	51-28-5	Sigma-Aldrich
Xylenesulfonate	1300-72-7	Chemtronica
2,6-Dichlorobenzamide	2008-58-4	Sigma-Aldrich
DNOC	534-52-1	Sigma-Aldrich
Dexpanthenol	81-13-0	Sigma-Aldrich
4-tert-Octylphenol	140-66-9	Sigma-Aldrich
1,3-Diphenylguanidine	102-06-7	Chemtronica
4-Nonylphenol	104-40-5	Sigma-Aldrich
Lidocaine	137-58-6	Sigma-Aldrich
1,3-Di-o-tolylguanidine	97-39-2	Sigma-Aldrich
N,N-Dimethyltetradecylamine	112-75-4	Chemtronica
Gemfibrozil	25812-30-0	Sigma-Aldrich
Carbamazepine-10,11-epoxide	36507-30-9	Sigma-Aldrich
Lamotrigine	84057-84-1	Sigma-Aldrich
Propranolol	318-98-9	Sigma-Aldrich
Indigo red	479-41-4	MedChemExpress (Fisher)
O-Desmethylvenlafaxine	93413-62-8	Sigma-Aldrich
Tramadol	36282-47-0	Sigma-Aldrich
Sodium Lauryl Sulfate	151-21-3	Sigma-Aldrich

Tri-n-butyl phosphate	126-73-8	Sigma-Aldrich
Metoprolol	56392-17-7	Sigma-Aldrich
Sotalol	959-24-0	Chemtronica
Estradiol	50-28-2	Sigma-Aldrich
Venlafaxine	99300-78-4	Sigma-Aldrich
Oxazepam	604-75-1	Sigma-Aldrich
Climbazole	38083-17-9	Sigma-Aldrich
Ethinyl Estradiol	57-63-6	Sigma-Aldrich
Hydrochlorothiazide	58-93-5	Sigma-Aldrich
Codeine	76-57-3	Sigma-Aldrich
Sertraline	79559-97-0	Sigma-Aldrich
Tebuconazole	107534-96-3	Sigma-Aldrich
Sulisobenzone	4065-45-6	Sigma-Aldrich
Ranitidine	66357-59-3	Sigma-Aldrich
Norfloxacin	70458-96-7	Sigma-Aldrich
Metconazole	125116-23-6	Sigma-Aldrich
Bis(2-ethylhexyl) phosphate	298-07-7	Sigma-Aldrich
Citalopram	59729-33-8	Sigma-Aldrich
Dimoxystrobin	149961-52-4	Sigma-Aldrich
Furosemide	54-31-9	Sigma-Aldrich
Ipconazole	125225-28-7	Chemtronica
Clotrimazole	23593-75-1	Sigma-Aldrich
Amoxicillin	26787-78-0	Sigma-Aldrich
Famoxadone	131807-57-3	Sigma-Aldrich
Losartan	114798-26-4	Sigma-Aldrich
Clindamycin	18323-44-9	Sigma-Aldrich
Irbesartan	138402-11-6	Sigma-Aldrich
Bicalutamide	90357-06-5	Sigma-Aldrich
Valsartan	137862-53-4	Sigma-Aldrich
Fexofenadine	83799-24-0	Sigma-Aldrich
Metaflumizone	139968-49-3	Sigma-Aldrich
Atorvastatin	134523-00-5	Sigma-Aldrich
Azithromycin	117772-70-0	Sigma-Aldrich
Dicyandiamide	461-58-5	Sigma-Aldrich
L-Glyceric acid	28305-26-2	Sigma-Aldrich
Hydroquinone	123-31-9	Sigma-Aldrich
Methyl sulfate	512-42-5	Sigma-Aldrich
Trifluoroacetic acid	76-05-1	Sigma-Aldrich
n-Nitrosomorpholine	59-89-2	Sigma-Aldrich
Benzimidazole	51-17-2	Sigma-Aldrich
Orcinol monohydrate	6153-39-5	Sigma-Aldrich
Melamine	108-78-1	Sigma-Aldrich
Quinoline	91-22-5	Fisher Scientific, Alfa Aesar

Metformin HCl	1115-70-4	Santa Cruz Biotechnology
2-Hydroxybenzimidazole	615-16-7	Sigma-Aldrich
1,3-benzothiazole	95-16-9	Fisher Scientific, Alfa Aesar
(−)-α-Pinene	7785-26-4	Sigma-Aldrich
4-hydroxybenzoic acid	99-96-7	Sigma-Aldrich
4-Nitrophenol	100-02-7	Sigma-Aldrich
Methamidophos	10265-92-6	Sigma-Aldrich
Ethephon	16672-87-0	VWR, Sigma Aldrich
1-Naphthol	90-15-3	Toronto Research Chemicals
2-Naphthol	135-19-3	Toronto Research Chemicals
Adipic acid	124-04-9	Sigma-Aldrich
5,6-Dimethylbenzimidazole	582-60-5	Sigma-Aldrich
Trifluoromethanesulfonic acid	1493-13-6	Sigma Aldrich
Phenylglyoxylic Acid	611-73-4	Toronto Research Chemicals
Paracetamol	103-90-2	Sigma Aldrich
Methyl 4-Hydroxybenzoate (Methyl Paraben)	99-76-3	Toronto Research Chemicals
4-Chloro-2-hydroxymethylphenol	5330-38-1	Fishersci, Alfa Aesar
2,5-Dichlorophenol	583-78-8	Toronto Research Chemicals
(−)-Nicotine	54-11-5	Sigma-Aldrich
Acesulfam, potassium	55589-62-3	VWR, TCI
Fenuron	101-42-8	Sigma-Aldrich
Phthalic acid	88-99-3	Sigma-Aldrich
Nor Harmane	244-63-3	Toronto Research Chemicals
Atrazine-desethyl-2-hydroxy	19988-24-0	Sigma-Aldrich
Metronidazole	443-48-1	Santa Cruz Biotechnology
Sulfanilamide	63-74-1	Fishersci, Alfa Aesar
Atrazine-desisopropyl	1007-28-9	Sigma-Aldrich
Cotinine	486-56-6	Toronto Research Chemicals
Memantine, HCl	41100-52-1	Chemtronica, Combi-blocks
Acetylsalicylic acid	50-78-2	Sigma / Sigma Aldrich
Propyl 4-Hydroxybenzoate (Propyl Paraben)	94-13-3	Sigma-Aldrich
2,4-dinitroaniline	97-02-9	Fishersci, Alfa Aesar
Adrenaline	51-43-4	Sigma-Aldrich
Simazine-2-hydroxy	2599-11-3	VWR, Dr Ehrenstorfer
Carboxymethyl-cyclohexanecarboxylic acid	67950-95-2	Sigma-Aldrich
Atrazine-desethyl	6190-65-4	Sigma-Aldrich
Azelaic acid	123-99-9	Sigma-Aldrich
Carbendazim	10605-21-7	Fishersci, Honeywell
DEET	134-62-3	Toronto Research Chemicals
4-Methyl Hippuric Acid	27115-50-0	Toronto Research Chemicals
Monoethyl Phthalate	2306-33-4	Toronto Research Chemicals
Caffeine	58-08-2	Santa Cruz Biotechnology
3,5,6 - Trichloro- 2- pyridinol	6515-38-4	Toronto Research Chemicals

Atrazine-2-hydroxy	2163-68-0	Sigma-Aldrich
N-Methyldodecylamine	7311-30-0	Sigma-Aldrich
N,N-Dimethyldecylamine N-oxide	2605-79-0	Sigma-Aldrich
Ibuprofen	15687-27-1	Sigma-Aldrich
N,N-Diethyl-4-methoxybenzamide	7465-86-3	Fishersci, Alfa Aesar
1-Naphthalenesulfonic acid	85-47-2	Chemtronica
2-Naphthalenesulfonic Acid Hydrate	76530-12-6	VWR, TCI
4-(Methylnitrosamino)-1-(3-pyridyl)-1-butanol	76014-81-8	Toronto Research Chemicals
Chrysoidine G	532-82-1	Santa Cruz Biotechnology
3-Phenoxybenzoic acid	3739-38-6	Sigma-Aldrich
Phenyl 4-hydroxybenzoate	17696-62-7	Sigma-Aldrich
Atrazine	1912-24-9	Sigma-Aldrich
6-Chloro-2,4-dinitroaniline	3531-19-9	Fishersci, Alfa Aesar
(+)-Nootkatone	4674-50-4	Sigma-Aldrich
2,4-Dichlorophenoxyacetic Acid	94-75-7	Toronto Research Chemicals
N-Acetyl-S-(N-methylcarbamoyl)-L-cysteine	103974-29-4	Toronto Research Chemicals
Carbofuran	1563-66-2	Santa Cruz Biotechnology
Prometon	1610-18-0	Sigma-Aldrich
Oxybenzone	131-57-7	Toronto Research Chemicals
Bisphenol A	80-05-7	Toronto Research Chemicals
Dimethoate	60-51-5	VWR, Honeywell
Icaridin	119515-38-7	Sigma-Aldrich, Ambeed
Dehydrocostus lactone	477-43-0	Sigma-Aldrich
Diuron	330-54-1	VWR, Sigma Aldrich
Carbamazepine	298-46-4	Santa Cruz Biotechnology
3,5-Di-tert-butyl-4-hydroxybenzyl alcohol	88-26-6	Chemtronica, Alfa Aesar
Monoisobutyl Phthalate	30833-53-5	Toronto Research Chemicals
Salbutamol	18559-94-9	Sigma-Aldrich
Bentazon	25057-89-0	Chemtronica, TCI
Cyanazine	21725-46-2	Sigma-Aldrich
(R,S)-Equol	94105-90-5	Toronto Research Chemicals
N-lauroylethanolamine	142-78-9	Sigma-Aldrich, Enamine
Rupestonic acid	115473-63-7	Ambinter
Diphenyl Phosphate	838-85-7	Toronto Research Chemicals
Sulfadiazine	68-35-9	Sigma-Aldrich
Sulfamethoxazole	723-46-6	Santa Cruz Biotechnology
Daidzein	486-66-8	Toronto Research Chemicals
Imidacloprid	138261-41-3	Fishersci, Honeywell
Palmitamide	629-54-9	Sigma-Aldrich, Ambeed
Dibutyl adipate	105-99-7	Sigma-Aldrich
2-bromo-4,6-dinitroaniline	1817-73-8	Fishersci, Alfa Aesar
Indigo	482-89-3	Merck
Pentachlorophenol	87-86-5	Toronto Research Chemicals

Atenolol	29122-68-7	Fisherci, Acros Organics
Dichlorophene	97-23-4	Sigma-Aldrich
Disperse yellow 3	2832-40-8	VWR, Sigma Aldrich
Clenbuterol hydrochloride	21898-19-1	Sigma-Aldrich
Sulfamethazine	57-68-1	Fisherci, Acros Organics
Metalaxyl	57837-19-1	VWR, Sigma Aldrich
Triclosan	3380-34-5	Toronto Research Chemicals
Benzoylecgonine	519-09-5	Sigma-Aldrich
Trimethoprim	738-70-5	VWR, MP Biomedicals
Thiamethoxam	153719-23-4	Santa Cruz Biotechnology
Mono(2-ethyl-5-hydroxyhexyl)Phthalate (Mixture of Diastereomerms)	40321-99-1	Toronto Research Chemicals
Tetradecyl sulfate sodium salt	1191-50-0	Sigma-Aldrich
Diclofenac sodium salt	15307-79-6	VWR, Sigma Aldrich
rac Enterolactone	78473-71-9	Toronto Research Chemicals
Bioallethrin	22431-63-6	VWR, Sigma Aldrich
Diazinon	333-41-5	Fisherci, Honeywell
Fluconazole	86386-73-4	Santa Cruz Biotechnology
Mono (5-carboxy-2-ethylpentyl)phthalate rac	40809-41-4	Toronto Research Chemicals
Triclocarban	101-20-2	Sigma-Aldrich
Malaoxon	1634-78-2	Sigma-Aldrich
Bis(1,3-dichloro-2-propyl)Phosphate	72236-72-7	Toronto Research Chemicals
Disperse Orange 1	2581-69-3	VWR, Sigma Aldrich
Chloramphenicol	56-75-7	VWR, MP Biomedicals
Malachite green chloride	569-64-2	VWR, Sigma Aldrich
Malathion	121-75-5	VWR, Sigma Aldrich
Ciprofloxacin	85721-33-1	Fisherci, Acros Organics
Propiconazole	60207-90-1	Fisherci, Honeywell
Chlorpyrifos	2921-88-2	VWR, Sigma Aldrich
Octocrylene	6197-30-4	Sigma-Aldrich
Crystal violet	548-62-9	Fisherci, MedChem Express
Sucralose	56038-13-2	LGC / Dr Ehrenstorfer
Tris(2-butoxyethyl) phosphate	78-51-3	Fisherci, Acros Organics
Pyrazosulfuron ethyl	93697-74-6	VWR, Sigma Aldrich
Cypermethrin	52315-07-8	Santa Cruz Biotechnology
Red 2G	3734-67-6	VWR, Sigma Aldrich
Indigo carmine	860-22-0	Unknown
Chlorantraniliprole	500008-45-7	VWR, Sigma Aldrich
3,3',5,5'-Tetrabromobisphenol A	79-94-7	Sigma-Aldrich
Reactive blue 4	13324-20-4	Fisherci, MedChem Express
Erythromycin	114-07-8	VWR, MP Biomedicals
Clarithromycin	81103-11-9	VWR, Alfa Aesar
Abamectin	71751-41-2	Santa Cruz Biotechnology
Perfluoro-n-butanoic acid	*	Wellington laboratories

Perfluoro-n-pentanoic acid	*	Wellington laboratories
Perfluoro-n-hexanoic acid	*	Wellington laboratories
Perfluoro-n-heptanoic acid	*	Wellington laboratories
Perfluoro-n-octanoic acid	*	Wellington laboratories
Perfluoro-n-nonanoic acid	*	Wellington laboratories
Perfluoro-n-decanoic acid	*	Wellington laboratories
Perfluoro-n-undecanoic acid	*	Wellington laboratories
Perfluoro-n-dodecanoic acid	*	Wellington laboratories
Perfluoro-n-tridecanoic acid	*	Wellington laboratories
Perfluoro-n-tetradecanoic acid	*	Wellington laboratories
Perfluoro-n-hexadecanoic acid	*	Wellington laboratories
Perfluoro-n-octadecanoic acid	*	Wellington laboratories
Potassium perfluoro-1-butanesulfonate	*	Wellington laboratories
Sodium perfluoro-1-hexanesulfonate	*	Wellington laboratories
Sodium perfluoro-1-octanesulfonate	*	Wellington laboratories
Sodium perfluoro-1-decanesulfonate	*	Wellington laboratories

*Purchased as mixture; no CAS number

Table S2. Labelled internal standards with related information.

Internal standard	CAS number	Supplier
Metformin-d6	1185166-01-1	Sigma-Aldrich
Acephate-(acetyl-d3)	2140327-70-2	Sigma-Aldrich
Propyl -d7 Paraben	1246820-92-7	Toronto Research Chemicals
Carbendazim-d4	291765-95-2	Sigma-Aldrich
DEET-d10	291759-05-2	Toronto Research Chemicals
Atrazine-2-hydroxy-d5	1276197-25-1	A2S certified
Atrazine-d5	163165-75-1	Sigma-Aldrich
2,4-Dichlorophenoxyacetic Acid-13C6	150907-52-1	Toronto Research Chemicals
Oxybenzone-d3	*	Toronto Research Chemicals
Diuron-d6	153466-65-0	Santa Cruz Biotechnology
Bisphenol A - 13C12	263261-65-0	Toronto Research Chemicals
Carbamazepine-d10	132183-78-9	Cerilliant
Diphenyl Phosphate -d10	93952-11-5	Toronto Research Chemicals
Pentachlorophenol-13C6	85380-74-1	Toronto Research Chemicals
Triclosan-13C6	*	Toronto Research Chemicals
Diclofenac -d4	1215576-01-4	Santa Cruz Biotechnology
Sucralose-d6	1459161-55-7	Toronto Research Chemicals
Perfluoro-n-[13C4]butanoic acid	Mixture*	Wellington laboratories
Perfluoro-n-[13C5]pentanoic acid		
Perfluoro-n-[1,2,3,4,6-13C5]hexanoic acid		
Perfluoro-n-[1,2,3,4-13C4]heptanoic acid		
Perfluoro-n-[13C8]octanoic acid		
Perfluoro-n-[13C9]nonanoic acid		
Perfluoro-n-[1,2,3,4,5,6-13C6]decanoic acid		
Perfluoro-n-[1,2,3,4,5,6,7-13C7]undecanoic acid		
Perfluoro-n-[1,2-13C2]dodecanoic acid		
Perfluoro-n-[1,2-13C2]tetradecanoic acid		
Sodium perfluoro-1-[2,3,4-13C3]butanesulfonate		
Sodium perfluoro-1-[1,2,3-13C3]hexanesulfonate		
Sodium perfluoro-1-[13C8]octanesulfonate		

*No CAS number

Sampling procedure

Samples were collected in pre-clean borosilicate flasks (100 or 50 mL). Cleaning procedure included dish washing followed by three rinses with milli-Q water, and finally rinsing with LC-MS grade methanol. Grab samples were taken according to following procedure created by Bonnefille et al. [13]: wearing nitrile gloves, the flask was opened ca. 10 cm under the water surface, capped and brought to the surface. Following this procedure, the flask was rinsed twice with the sample before collecting the sample. Ca. 80 % of the flask volume was filled to avoid cracking of the flasks during freezing (-20 °C) for storage.

Ground waters were sampled by Swedish Geological Survey (SGU) by two different approaches 1) pumping ground water (40 mL) from a monitoring well through a silicone tube into a 50 mL borosilicate flask, or 2) grab sample from a natural spring following the procedure described above. Ground waters analysed in the current study was a mixture of ground waters, due to low sample volume, collected with both approaches collected in Öjesjöv (monitoring well; 08.04.2021; coordinates 59.86291, 16.27758), Kappa källa (natural spring; 08.04.2021, coordinates 59.778480, 17.08572) and Vimmerby (two sampling sites; natural springs; 02.05.2021, coordinates 57.80004, 15.74051 and 57.51488, 15.839603).

Wastewater was collected by Stockholm Waste and Water (SVoA) as a one-week composite sample by mixing wastewaters continuously pumped through a PCV tube over 24 hours. The sample were collected week 23 2021, and consisted of household waters ingoing to a major wastewater treatment plant (WWTP) in Stockholm. The wastewater analysed was therefore not treated at a WWTP.

Surface water was collected as a grab sample in Mälaren lake in Stockholms county (coordinates 59.34345, 18.00267), 06.09.2022 following the grab sample procedure described above. Blank sample analysed acted as a transport blank, and consisted of LC-MS optima grade water which were brought along for the sampling of the surface water.

All samples were stored with cooling elements in insulated boxes short term until storage in freezer (-20 °C).

Sample preparation

Samples stored in the freezer (-20 °C) were thawed (room temperature) the same day as the samples were prepared. Sample preparation followed a workflow from Bonnefille et al. [13] with slight modifications. 11 mL of the vortexed sample was transferred to a furnaced glass test tube and spiked with 28 isotopically labelled internal standards to a total concentration of 200 ng/L (Table S2). After vortexing, the sample was filtered (0.2 µm, Sartorius Minisart ™, 15 mm) into a new furnaced glass test tube, and 10 mL was transferred to an injection vial. *Spiked* samples were spiked with a mixture of 216 native standards (Table S1) dissolved in MeOH to a total concentration of 100 ng/L (assuming the analyte was not present in sample). Finally, all samples were spiked with two isotopically labelled internal standards to a total concentration of 200 ng/L (Table S2).

LC-MS optima spiked matrix was prepared by transferring 10 mL LC-MS optima grade water to an injection vial. 30 isotopically labelled internal standards were spiked (200 ng/L) in addition to 216 native standards to a total concentration of 100 ng/L (table S1 and S2). A blank sample to account for background corrections for this sample type was prepared by transferring 10 mL LC-MS grade water into an injection vial.

Online SPE-LC-HRMS

The samples were injected in randomized order within each acquisition method, except all wastewater injections were positioned at the end of the sequence to avoid sensitivity loss early in the sequence due to the complex sample matrix. The online SPE-UPLC procedure followed the procedure from Bonnefille et al. [13] with slight modifications. 1.1 mL of the sample was injected into a 1 mL sample loop, and was further loaded on a hydrophilic-lipophilic balance (HLB) online-SPE column (Waters Oasis HLB, 15 µm, 2.1 x 20 mm, 80 Å) for 2.2 minutes, at a flow rate of 0.5 mL/min. The online-SPE column was then back-flushed and directed the sample to the analytical column throughout the LC gradient (from 2.2 min to 19 minutes). Reversed phased separation was achieved on a Waters Acquity UPLC BEH C18 column (1.7 µm, 2.1 x 100 mm, 130 Å) with corresponding guard column (Waters Acquity UPLC BEH C18 VanGuard Precolumn, 1.7 µm, 2.1 x 5 mm, 130 Å). Mobile phase A (MP A) was 1 mM ammonium fluoride in LC-MS grade water, and mobile phase B (MP B) was LC-MS grade methanol (MeOH). Column temperature was set to 40 °C, and the flow rate to 0.4 mL/min. The gradient started with 15 % MeOH and increased linearly to 80 % MeOH from 2.2 min to 14 min, followed by a steeper linear increase to 100 % MeOH to 16 minutes. 100 % MeOH was held until 19 minutes, then the column was re-equilibrated in 15 % MeOH to the end of sequence (22 minutes), continuing until the online-SPE back flushing at 2.2 min for the following injection.

HRMS data was obtained with a Q Exactive Orbitrap HF-X (Thermo Fischer Scientific), calibrated in the relevant ionization mode the same day as the sequence was started. Electrospray ionization (ESI) was applied for ionization in both positive and negative ionization mode in separate injections. For comparison between data independent acquisition (DIA) and data dependent acquisition (DDA), the HRMS was operated with two different data acquisition methods. In DIA, the HRMS alternated between six scan events, first a MS1 full-scan (m/z 90-1050; 120 000 resolution FWHM at 200 m/z), followed by five MS2 DIA scans with isolation windows of variable precursor m/z ranges (m/z 90-150, 145-205, 200-300, 295-505, 500-1000; 30 000 resolution FWHM at 200 m/z). In DDA, the HRMS alternated between a MS1 full-scan (similar to DIA method) and Top10 most intense precursor MS2 scans (30 000 resolution FWHM at 200 m/z, 10 s dynamic exclusion).

Manual inspection of the data

Manual inspections of the data were performed in Thermo Xcalibur Qual browser (Thermo Fisher Scientific Inc., v. 4.2.47), TraceFinder™ (Thermo Fisher Scientific Inc., v. 5.1 SP3), and MS-Dial (v. 4.80).

Method limitations

Out of 216 chemicals spiked, 191 chemicals were confirmed recovered by manual inspections of the raw data. 25 chemicals were not recovered due to method limitations, due to the chemical's physical properties was outside the method coverage (marked in grey in Table S1).

Data pre-treatment in MS-DIAL (v. 4.80)

Table S3. Applied MS-DIAL parameters.

**Data collection**	
MS1 tolerance	0.001 Da
MS2 tolerance	0.003 Da
RT begin	2 min
RT end	18 min
MS1 mass range begin	90 Da
MS1 mass range end	1050 Da
MS/MS mass range begin	90 Da
MS/MS mass range end	1050 Da
Maximum charged number	2
Consider Cl and Br elements	Yes
**Peak detection**	
Min peak height	10000 amplitude
Mass slice width	0.07 Da
Smoothing method	Linear weighted moving average
Smoothing level	5 scan
Min peak width	8 scan
**MS2Dec**	
Sigma window value	1
MS/MS abundance cut off	0 amplitude
Exclude after precursor ion	Yes
Keep the isotopic ions until	5 Da
Keep the isotopic ions w/o MS2Dec	Yes
**Identification**	
MSP file:	MassBank Europe data release 15.12.2022
RT tolerance	100 min
MS1 accurate mass tolerance	0.002 Da
MS2 accurate mass tolerance	0.005 Da
Identification score cut off	60 %
Use RT for scoring	No
Use RT for filtering	No
Post identification	None
RT tolerance	0.2 min
Accurate mass tolerance	0.002 Da
Identification score cut off	85 %
Rel. abundance cut off	0 %
Only report the top hit	No
**Adduct**	
Negative ionization mode	[M-H]-

Positive ionization mode	[M+H]+, [M+Na]+, [M+NH4]+
**Alignment**	
RT tolerance	0.75 min
MS1 tolerance	0.003 Da
RT factor	0.5
MS1 factor	0.5
N% detected in at least one group	100 %
Remove features based on blank information	Sample max/blank average
Fold change	5
Keep ref.matched features	Yes
keep suggested w/o MS2 features	No
Keep removable features and assign the tag	Yes
Gap filling by compulsion	Yes

Results

Table S4. Aligned detected features in MS-DIAL after blank filtration (N=100 %).

**Sample, method**	**Total number of MS1 features detected**	**MS2 acquired (total number)**	**Proportion of MS1 features with MS2 acquired (%)**
LC-MS optima grade water spiked, DDA ESI+	1,256	521	41.5
LC-MS optima grade water spiked, DIA ESI+	979	978	99.9
LC-MS optima grade water spiked, DDA ESI-	1,093	479	43.8
LC-MS optima grade water spiked, DIA ESI-	910	908	99.8
Groundwater spiked, DDA ESI+	2,232	559	25.0
Groundwater spiked, DIA ESI+	2,721	2,720	100.0
Groundwater spiked, DDA ESI-	2,449	760	31.0
Groundwater spiked, DIA ESI-	2,732	2,731	100.0
Surface water spiked, DDA ESI+	2,565	614	23.9
Surface water spiked, DIA ESI+	2,859	2,856	99.9
Surface water spiked, DDA ESI-	2,564	775	30.2
Surface water spiked, DIA ESI-	2,850	2,845	99.8
Wastewater spiked, DDA ESI+	8,844	3,405	38.5
Wastewater spiked, DIA ESI+	11,696	11,623	99.4
Wastewater spiked, DDA ESI-	5,686	2,822	49.6
Wastewater spiked, DIA ESI-	7,470	7,444	99.7

Table S5. Recovery standards (N=191) with mass (Da), average retention time in LC-MS water (RT; min), and information of spectral library matches with MassBank Europe. Spectral library matches are marked "yes" if first ranked match in MS-DIAL is correct for standards spiked at 100 ng/L in LC-MS water, groundwater, surface water and wastewater, with DDA and DIA methods in electrospray ionization in negative (-) and positive mode (+).

Analytical standard	Mass (Da)	RT (min)	DDA method: MassBank Europe spectral library matches. For standards spiked in:								DIA method: MassBank Europe spectral library matches. For standards spiked in:							
			LC-MS water		Ground -water		Surface water		Waste- water		LC-MS water		Ground -water		Surface water		Waste- water	
			-	+	-	+	-	+	-	+	-	+	-	+	-	+	-	+
Aniline	93.0579	3.85																
ε-Caprolactam	113.0841	4.92																
1H-Benzotriazole	119.0484	5.58	yes	yes						yes	yes	yes	yes	yes	yes	yes		yes
4-Methyl-1H-benzotriazole	133.0640	7.39	yes	yes		yes			yes		yes	yes		yes	yes	yes	yes	
5-Methyl-1H-benzotriazole	133.0640	7.39	yes	yes		yes			yes			yes	yes	yes	yes	yes	yes	
4-Toluenesulfonamide	171.0354	5.84	yes								yes				yes			
Gabapentin	171.1259	4.26										yes						
p-Toluenesulfonic acid	172.0194	4.14			yes								yes				yes	
2,4-Dinitrophenol	184.0120	7.04																
Xylenesulfonate	186.0351	5.15																
2,6-Dichlorobenzamide	188.9748	5.22		yes								yes						
DNOC	198.0277	6.13	yes								yes		yes		yes			
Dexpanthenol	230.1307	3.90	yes	yes					yes	yes							yes	
4-tert-Octylphenol	206.1671	15.13																
1,3-Diphenylguanidine	211.1110	6.08	yes	yes							yes	yes						
Lidocaine	234.1732	8.78		yes								yes						

1,3-Di-o-tolylguanidine	239.1423	7.26																
N,N-Dimethyltetra-decylamine	241.2770	15.86		yes		yes		yes		yes		yes		yes		yes		yes
Gemfibrozil	250.1569	14.18	yes								yes							
Carbamazepine-10,11-epoxide	252.0899	8.33				yes		yes		yes				yes		yes		yes
Lamotrigine	255.0079	7.22		yes		yes		yes		yes		yes		yes		yes		yes
Propranolol	259.1572	9.23		yes		yes						yes						
O-Desmethyl-venlafaxine	263.1885	6.50	yes	yes		yes					yes	yes				yes		
Tramadol	263.1885	6.81																
Tri-n-butyl phosphate	266.1647	14.71																
Metoprolol	267.1834	6.90		yes								yes						yes
Sotalol	272.1195	3.62	yes	yes		yes		yes			yes	yes		yes		yes		
Venlafaxine	277.2042	8.79		yes				yes				yes		yes				
Oxazepam	286.0509	11.06	yes	yes	yes	yes		yes		yes	yes	yes	yes		yes			
Climbazole	292.0979	12.90	yes	yes	yes		yes		yes	yes	yes	yes						yes
Ethinyl Estradiol	296.1776	12.10																
Hydrochlorothiazide	296.9645	3.84	yes								yes						yes	
Codeine	299.1521	5.11		yes														
Sertraline	305.0738	12.43		yes								yes						
Tebuconazole	307.1451	13.79		yes		yes		yes				yes						yes
Sulisobenzone	308.0355	7.43	yes	yes	yes		yes		yes	yes	yes	yes	yes		yes	yes	yes	yes
Ranitidine	314.1413	3.92	yes	yes							yes	yes						
Norfloxacin	319.1332	6.91																
Metconazole	319.1451	14.16		yes		yes		yes				yes		yes		yes		
Bis(2-ethylhexyl) phosphate	322.2273	15.18	yes	yes	yes						yes	yes			yes			

Citalopram	324.1638	9.39		yes								yes		yes		yes		
Dimoxystrobin	326.1630	13.55		yes		yes		yes				yes						
Furosemide	330.0077	6.81	yes		yes				yes		yes		yes		yes		yes	
Ipconazole	333.1608	14.76		yes		yes		yes				yes		yes				
Clotrimazole	344.1080	14.56										yes						
Amoxicillin	365.1045	3.45																
Famoxadone	374.1267	14.07	yes															
Losartan	422.1622	10.77	yes	yes	yes	yes	yes	yes	yes	yes	yes	yes	yes	yes	yes	yes	yes	yes
Clindamycin	424.1799	12.98		yes				yes				yes				yes		yes
Irbesartan	428.2325	11.65	yes	yes	yes	yes	yes	yes	yes	yes	yes	yes	yes	yes	yes	yes	yes	yes
Bicalutamide	430.0610	11.47	yes	yes	yes		yes		yes		yes		yes		yes			
Valsartan	435.2270	10.46	yes	yes					yes			yes						
Fexofenadine	501.2879	10.45	yes	yes		yes		yes	yes	yes	yes	yes		yes		yes	yes	yes
Metaflumizone	506.1177	15.34																
Atorvastatin	558.2530	12.53	yes	yes	yes	yes	yes	yes	yes	yes	yes	yes	yes		yes		yes	
Azithromycin	748.5085	9.64										yes						
Hydroquinone	110.0368	9.24																
n-Nitrosomorpholine	116.0586	3.86																
1H-Benzimidazole	118.0531	5.52	yes	yes	yes							yes	yes	yes	yes			
orcinol monohydrate	124.0524	5.13																
Melamine	126.0654	3.19		yes														
Quinoline	129.0578	8.37		yes		yes		yes				yes		yes		yes		yes
Metformin HCl	129.1014	3.31		yes		yes		yes		yes		yes		yes		yes		yes
2-Hydroxy-benzimidazole	134.0480	5.42		yes								yes						

1,3-benzothiazole	135.0143	8.30		yes														
4-Nitrophenol	139.0269	6.71	yes								yes							
Methamidophos	141.0013	3.60																
1-Naphthol	144.0575	10.33									yes							
2-Naphthol	144.0575	10.01	yes								yes		ye s		ye s		yes	
5,6-Dimethyl-benzimidazole	146.0844	8.49		yes		yes		yes				yes	ye s	yes	ye s	yes		yes
Trifluoromethanesulfonic acid	149.9599	3.36	yes								yes		ye s		ye s		yes	
Paracetamol	151.0633	3.96	yes						yes	yes		yes					yes	yes
Methyl 4-Hydroxybenzoate	152.0473	7.45	yes								yes		ye s		ye s		yes	
4-Chloro-2-hydroxymethylphenol	158.0135	7.28																
2,5-Dichlorophenol	161.9639	10.95																
(−)-Nicotine	162.1157	3.70		yes								yes						
Acesulfam, potassium	162.9939	3.38	yes				yes		yes		yes		ye s		ye s		yes	
Fenuron	164.0950	6.12	yes	yes		yes		yes				yes		yes		yes		yes
Nor Harmane	168.0688	9.46									yes		ye s		ye s		yes	
Atrazine-desethyl-2-hydroxy	169.0964	4.31	yes	yes							yes	yes						
Metronidazole	171.0644	4.38	yes	yes		yes		yes				yes		yes		yes		
Sulfanilamide	172.0307	3.77	yes								yes	yes						
Atrazine-desisopropyl	173.0468	5.55		yes								yes		yes		yes		
Cotinine	176.0950	4.78		yes						yes		yes		yes		yes		yes
Memantine, HCl	179.1674	9.57		yes								yes						
Propyl 4-Hydroxybenzoate	180.0786	10.75	yes								yes		ye s		ye s		yes	

2,4-dinitroaniline	183.0280	7.61	yes								yes		yes		yes			
Simazine-2-hydroxy	183.1120	6.12	yes	yes							yes	yes		yes		yes		yes
Atrazine-desethyl	187.0625	7.11		yes								yes		yes		yes		
Carbendazim	191.0695	7.07		yes		yes		yes		yes		yes		yes		yes		yes
DEET	304.1011	10.77		yes		yes		yes		yes		yes		yes		yes		yes
4-Methyl Hippuric Acid	193.0739	4.97						yes										
Monoethyl Phthalate	194.0579	4.52									yes						yes	
Caffeine	194.0804	5.06		yes		yes				yes		yes		yes		yes		yes
3,5,6 - Trichloro- 2-pyridinol	196.9202	8.87	yes								yes		yes		yes		yes	
Atrazine-2-hydroxy	197.1277	7.79	yes	yes							yes	yes						
N-Methyldodecylamine	199.2300	14.23		yes		yes				yes		yes		yes				yes
N,N-Dimethyl-decylamine N-oxide	201.2093	12.96		yes		yes		yes		yes		yes		yes		yes		
Ibuprofen	206.1307	12.03																
N,N-Diethyl-4-methoxybenzamide	207.1259	9.85																
1-Naphthalene-sulfonic acid	208.0194	5.47																
2-Naphthalene-sulfonic Acid	208.0194	5.89	yes				yes				yes		yes		yes		yes	
4-(Methylnitros-amino)-1-(3-pyridyl)-1-butanol	209.1164	5.00																
Chrysoidine G	212.1062	10.54		yes														
3-Phenoxybenzoic acid	214.0630	11.49	yes								yes						yes	
Phenyl 4-hydroxybenzoate	214.0630	11.49																
Atrazine	215.0938	10.47		yes		yes		yes				yes		yes		yes		yes

6-Chloro-2,4-dinitroaniline	216.9890	9.32	yes								yes		yes		yes			
(+)-Nootkatone	218.1671	14.15		yes		yes		yes				yes		yes		yes		
2,4-Dichlorophen-oxyacetic Acid	219.9694	8.01											yes		yes		yes	
N-Acetyl-S-(N-methylcarbamoyl)-L-cysteine	220.0518	3.23																
Carbofuran	221.1052	9.24		yes		yes		yes		yes		yes		yes		yes		yes
prometon	225.1590	11.56		yes		yes		yes		yes		yes		yes		yes		yes
Oxybenzone	228.0786	13.20	yes	yes		yes				yes	yes	yes		yes		yes		yes
Bisphenol A	228.1150	11.18	yes		yes		yes				yes		yes		yes			
Dimethoate	228.9996	6.43		yes		yes		yes		yes		yes		yes		yes		
Icaridin	229.1678	11.49		yes								yes		yes		yes		yes
Dehydrocostus lactone	191.1310	13.39		yes		yes		yes				yes						
Diuron	232.0170	10.87	yes	yes	yes	yes	yes	yes	yes	yes	yes	yes	yes	yes	yes	yes	yes	
Carbamazepine	236.0950	10.04		yes		yes		yes		yes		yes		yes		yes		yes
3,5-Di-tert-butyl-4-hydroxybenzyl alcohol	236.1776	13.29	yes		yes				yes		yes		yes					
salbutamol	239.1521	3.70	yes	yes							yes	yes						
Bentazon	240.0569	5.18			yes		yes				yes		yes		yes			
Cyanazine	240.0890	8.69		yes		yes		yes				yes		yes				
(R,S)-Equol	242.0943	9.61	yes		yes		yes				yes		yes		yes			
N-lauroylethanolamine	243.2198	14.88	yes	yes	yes	yes	yes	yes	yes	yes	yes	yes	yes	yes	yes	yes		yes
Rupestonic acid	248.1412	8.54									yes							

Diphenyl Phosphate	250.0395	8.24	yes	yes	yes	yes	yes	yes	yes	yes	yes	yes	yes	yes	yes	yes	yes	yes
Sulfadiazine	250.0525	3.76	yes	yes		yes		yes				yes		yes		yes		
Sulfamethoxazole	253.0521	5.07	yes	yes	yes	yes	yes	yes	yes	yes	yes	yes			yes			yes
Daidzein	254.0579	8.98		yes	yes	yes		yes		yes	yes	yes						yes
Imidacloprid	255.0523	5.94	yes	yes		yes	yes	yes	yes	yes	yes	yes	yes	yes	yes	yes	yes	yes
Palmitamide	255.2562	16.61		yes														
Dibutyl adipate	258.1831	14.74		yes														
2-bromo-4,6-dinitroaniline	260.9385	9.81																
Indigo	262.0742	13.44																
Pentachlorophenol	263.8470	12.48	yes		yes		yes		yes		yes							
Atenolol	266.1630	3.70	yes	yes		yes		yes			yes	yes		yes		yes		
Dichlorophene	268.0058	13.49	yes		yes		yes		yes		yes		yes		yes		yes	
Disperse yellow 3	269.1164	13.73		yes							yes	yes						
Clenbuterol hydrochloride	276.0796	6.59		yes		yes		yes				yes		yes		yes		
Sulfamethazine	278.0837	5.22	yes	yes	yes	yes	yes	yes		yes	yes	yes	yes	yes		yes		
Metalaxyl	279.1471	10.92		yes		yes		yes		yes		yes		yes		yes		yes
Triclosan	287.9512	14.91																
benzoylecgonine	289.1314	5.83		yes		yes		yes		yes		yes		yes		yes		yes
Trimethoprim	290.1379	5.91	yes	yes								yes						
Thiamethoxam	291.0193	5.04		yes		yes		yes		yes		yes		yes		yes		
Mono(2-ethyl-5-hydroxyhexyl) Phthalate	294.1467	8.92	yes		yes				yes		yes		yes		yes			
Diclofenac sodium	295.0167	11.44	yes	yes	yes	yes		yes	yes	yes	yes	yes	yes	yes	yes	yes	yes	yes

rac Enterolactone	298.1205	9.70	yes	yes	yes		yes		yes	yes	yes		ye s		ye s		yes	yes
Bioallethrin	302.1882	15.34		yes		yes		yes				yes						
Diazinon	205.1314	13.86		yes		yes		yes		yes		yes				yes		yes
Fluconazole	306.1041	6.73	yes	yes	yes	yes	yes	yes	yes	yes	yes	yes	ye s		ye s	yes	yes	yes
Mono (5-carboxy-2-ethylpentyl) phthalate	308.1260	7.31							yes									
Triclocarban	313.9781	14.69									yes	yes						
Malaoxon	314.0589	9.49		yes		yes		yes		yes		yes		yes		yes		
Bis(1,3-dichloro-2-propyl)Phosphate	317.9149	9.34																
Disperse Orange 1	318.1117	15.74		yes	yes	yes	yes	yes				yes		yes		yes		
Chloramphenicol	322.0123	7.15	yes	yes	yes		yes				yes	yes	ye s		ye s			
Malathion	330.0361	12.35	yes	yes						yes	yes	yes						
Ciprofloxacin	331.1332	6.88																
Propiconazole	341.0698	13.94		yes		yes		yes				yes		yes		yes		yes
Chlorpyrifos	348.9263	15.47						yes				yes		yes		yes		
Octocrylene	361.2042	16.07												yes				yes
Sucralose	396.0146	5.56															yes	
Tris(2-butoxyethyl) phosphate	398.2433	15.10		yes		yes		yes		yes		yes		yes		yes		yes
Pyrazosulfuron ethyl	414.0958	8.71	yes	yes	yes		yes				yes	yes						
Cypermethrin	415.0742	16.02																
Red 2G	465.0301	6.00																
Chlorantraniliprole	480.9708	11.44	yes	yes	yes	yes	yes							yes	ye s	yes		
3,3',5,5'-Tetra-bromobisphenol A	539.7571	14.90	yes		yes		yes				yes		ye s		ye s			
Erythromycin	733.4612	11.24		yes								yes		yes				

Clarithromycin	747.4769	12.41		yes								yes				yes		yes
Abamectin	872.4922	16.33																
Perfluoro-n-butanoic acid	213.9865	5.41																
Perfluoro-n-pentanoic acid	263.9833	8.06	yes		yes		yes		yes		yes		yes		yes			
Perfluoro-n-hexanoic acid	313.9801	10.10	yes		yes		yes		yes		yes		yes		yes		yes	
Perfluoro-n-heptanoic acid	363.9769	11.47	yes		yes		yes		yes		yes		yes		yes		yes	
Perfluoro-n-octanoic acid	413.9737	12.48	yes		yes		yes		yes		yes		yes		yes		yes	
Perfluoro-n-nonanoic acid	463.9705	13.30	yes		yes		yes		yes		yes		yes		yes		yes	
Perfluoro-n-decanoic acid	513.9673	13.98	yes		yes		yes		yes		yes		yes		yes		yes	
Perfluoro-n-undecanoic acid	563.9641	14.56	yes		yes		yes		yes		yes		yes		yes		yes	
Perfluoro-n-dodecanoic acid	613.9609	15.04	yes		yes						yes		yes		yes		yes	
Perfluoro-n-tridecanoic acid	663.9577	15.40	yes		yes		yes		yes		yes		yes				yes	
Perfluoro-n-tetradecanoic acid	713.9545	15.66	yes		yes		yes		yes		yes		yes		yes		yes	
Perfluoro-n-hexadecanoic acid	813.9482	16.04																
Perfluoro-n-octadecanoic acid	913.9418	16.31																
Perfluoro-1-butanesulfonate	299.9503	8.71	yes		yes		yes		yes		yes		yes		yes		yes	
Perfluoro-1-hexanesulfonate	399.9439	11.61																
Perfluoro-1-octanesulfonate	499.9375	13.34	yes		yes		yes		yes		yes						yes	
Perfluoro-1-decanesulfonate	599.9311	14.56	yes		yes		yes		yes		yes		yes		yes		yes	
Total per ionization mode			81	99	45	58	38	54	39	42	78	96	52	58	53	57	43	44

Total both ionization modes	138	88	81	65	139	98	96	77

Table S6. Annotated structures (reference chemicals and level 2a) prioritized with MS2Tox (high toxicity + high CombinedConfidence) for case-study. Table 2 with InChlKeys.

Sample matrix*	MS2Tox predicted LC_{50} (mg/L)	Combined confidence	Compound (Confidence Level[49])	InChlKey	Classification	CompTox predicted LC_{50} (mg/L), experimental when available	LC50 prediction with MS2Tox from annotation (mg/L)
Wastewater^{DDA}	0.27	Correct formula	Rac Enterolactone (1)	HVDGDHBAMCBBLR-WMLDXEAASA-N	Natural product, metabolite	0.97	0.27
Wastewater^{DIA}	0.24	Correct formula	N,N-Dimethyl tetradecylamine (1)	SFBHPFQSSDCYSL-UHFFFAOYSA-N	PCPs (antistatic), Industrial	0.22 (exp 0.18)	3.40
Surface water^{DIA}	0.78	Incorrect formula	Lamotrigine (1)	PYZRQGJRPPTADH-UHFFFAOYSA-N	Pharmaceutica	7.58	3.94
Wastewater^{DIA}	1.36	Incorrect formula	Losartan (1)	PSIFNNKUMBGKDQ-UHFFFAOYSA-N	Pharmaceutica	0.061 (nearest neighbour only)	0.70
Surface water^{DIA}	2.07	Incorrect formula	Perfluorobutane sulfonate (1)	JGTNAGYHADQMCM-UHFFFAOYSA-M	PFAS	25.58	3.47
Wastewater^{DIA}	2.19	Correct formula	N-lauroylethanolamine (1)	QZXSMBBFBXPQHI-UHFFFAOYSA-N	PCPs (Surfactant, foaming agent)	4.66	13.4
Ground water^{DIA}	2.38	Correct formula	Diphenyl Phosphate (1)	ASMQGLCHMVWBQR-UHFFFAOYSA-N	Industrial	0.56	4.01
Wastewater^{DDA}	0.17	3.85	Vicenin 2 (2a)	FIAAVMJLAGNUKW-UHFFFAOYSA-N	Natural product	-	0.17
Wastewater^{DDA}	0.22	3.6	Triethylene glycol dibenzoate (2a)	AHSGHEXYEABOKT-UHFFFAOYSA-N	Plasticizer, adhesive	2.35	1.73
Wastewater$^{DDA, DIA}$	0.93, 3.6	3.91, 3.82	Diosgenin (2a)	WQLVFSAGQJTQCK-VKROHFNGSA-N	Natural product	-	0.10
Wastewater^{DDA}	1.8	3.84	Hesperetin (2a)	AIONOLUJZLIMTK-AWEZNQCLSA-N	Natural product	0.74	1.80
Wastewater^{DDA}	0.78	3.35	L-Proline (2a)	ONIBWKKTOPOVIA-BYPYZUCNSA-N	Natural product, metabolite	451.79	8.34
Wastewater^{DDA}	2.4	3.87	Mycophenolic Acid (2a)	HPNSFSBZBAHARI-UHFFFAOYSA-N	Natural product	-	2.40
Wastewater^{DDA}	1.9	4.09	N-Acetyl Mesalazine (2a)	GEFDRROBUCULOD-UHFFFAOYSA-N	Pharmaceutical metabolite	123.43	1.90
Surface water^{DIA}	1.3	3.37	Etilefrine (2a)	SQVIAVUSQAWMKL-UHFFFAOYSA-N	Pharmaceutica	74.89	6.42
Wastewater^{DIA}	3.2	3.54	3-Dehydrocholic acid (2a)	OEKUSRBIIZNLHZ-YXRVOZSUSA-N	Metabolite	-	26.06

Wastewater[DIA]	1.7	4.29	4-Pyridoxate (2a)	HXACOUQIXZGNBF-UHFFFAOYSA-N	Natural product, metabolite	375.58	1.66
Wastewater[DIA]	3.6	3.65	N-Oleyl-Isoleucine (2a)	TYWACVZIFBZIQZ-BSFUXZNJSA-N	Natural product, metabolite	0.28	49.48
Wastewater[DIA]	2.1	3.69	2,6-Di-tert-butyl-1,4-benzoquinone (2a)	RDQSIADLBQFVMY-UHFFFAOYSA-N	Natural product	3.47	8.65
Wastewater[DIA]	2.2	3.75	Ritalinic acid (2a)	INGSNVSERUZOAK-UHFFFAOYSA-N	Pharmaceutical metabolite	16.95	21.85

*MS^2 acquisition method

Table S7. Annotated structures (reference chemicals and level 2a) prioritized with MS2Tox (low toxicity + high CombinedConfidence) for case-study, predicted fish LC50 with belonging CombinedConfidence for non-target features (2a) or formula prediction information for reference chemicals (1), classification, and 96 hours fathead minnow LC50 predicted by CompTox Chemicals Dashboard, and by MS2Tox from annotated structure. InChlKeys are provided in Table S6.

Sample matrix*	MS2Tox predicted LC_{50} (mg/L)	Combined confidence	Compound (Confidence Level)	InChlKey	Classification	CompTox predicted LC_{50} (mg/L)	LC50 prediction with MS2Tox from annotation (mg/L)
Surface- and Wastewater[DIA]	129.84; 278.70	Correct formula	Metformin (1)	OETHQSJEHLVLGH-UHFFFAOYSA-N	Pharmaceutical	-	248.8
Ground water[DIA]	246.89	3.31	Lupinine (2a)	HDVAWXXJVMJBAR-VHSXEESVSA-N	Natural product	127.94	75.00
Ground- and Surface water[DIA]	265.63	3.61; 3.45	tert-Butyl methacrylate (2a)	SJMYWORNLPSJQO-UHFFFAOYSA-N	Industrial chemical	11.94	289.09
Surface water[DIA]	336.76	3.83	Nivalenol (2a)	UKOTXHQERFPCBU-UHFFFAOYSA-N	Fungicide	2.71	16.72
Wastewater[DDA]	1,650.40	3.90	Isoleucine (2a)	AGPKZVBTJJNPAG-UHFFFAOYSA-N	Amino acid	331.75	25.51
Wastewater[DDA]	185.99	3.60	Ectoine (2a)	WQXNXVUDBPYKBA-YFKPBYRVSA-N	Metabolite	-	57.78
Wastewater[DDA]	273.75	4.38	Acetylcholine (2a)	OIPILFWXSMYKGL-UHFFFAOYSA-N	Metabolite	43.86 (nearest neighbour only)	345.61

Wastewater[DIA]	1,255.82	3.58	4-Methylvaleric acid	FGKJLKRYENPLQH-UHFFFAOYSA-N	Natural product	97.00	350.74
Wastewater[DIA]	396.75	3.57	Decanoic acid (2a)	GHVNFZFCNZKVNT-UHFFFAOYSA-N	Natural product	3.7	167.59
Wastewater[DIA]	274.64	3.78	6-Hydroxy nicotinic acid (2a)	BLHCMGRVFXRYRN-UHFFFAOYSA-N	Natural product /metabolite	244.82	98.62
Wastewater[DIA]	218.47	3.76	5-Methoxysalicylic acid (2a)	IZZIWIAOVZOBLF-UHFFFAOYSA-N	Natural product /metabolite	96.62	218.47
Wastewater[DIA]	209.84	3.49	Trans-2-Hexenal (2a)	MBDOYVRWFFCFHM-SNAWJCMRSA-N	Natural product	9.27	577.67
Wastewater[DIA]	163.81	3.90	Hexanedioic acid (2a)	WNLRTRBMVRJNCN-UHFFFAOYSA-N	Metabolite	97.01	163.81

*MS^2 acquisition method

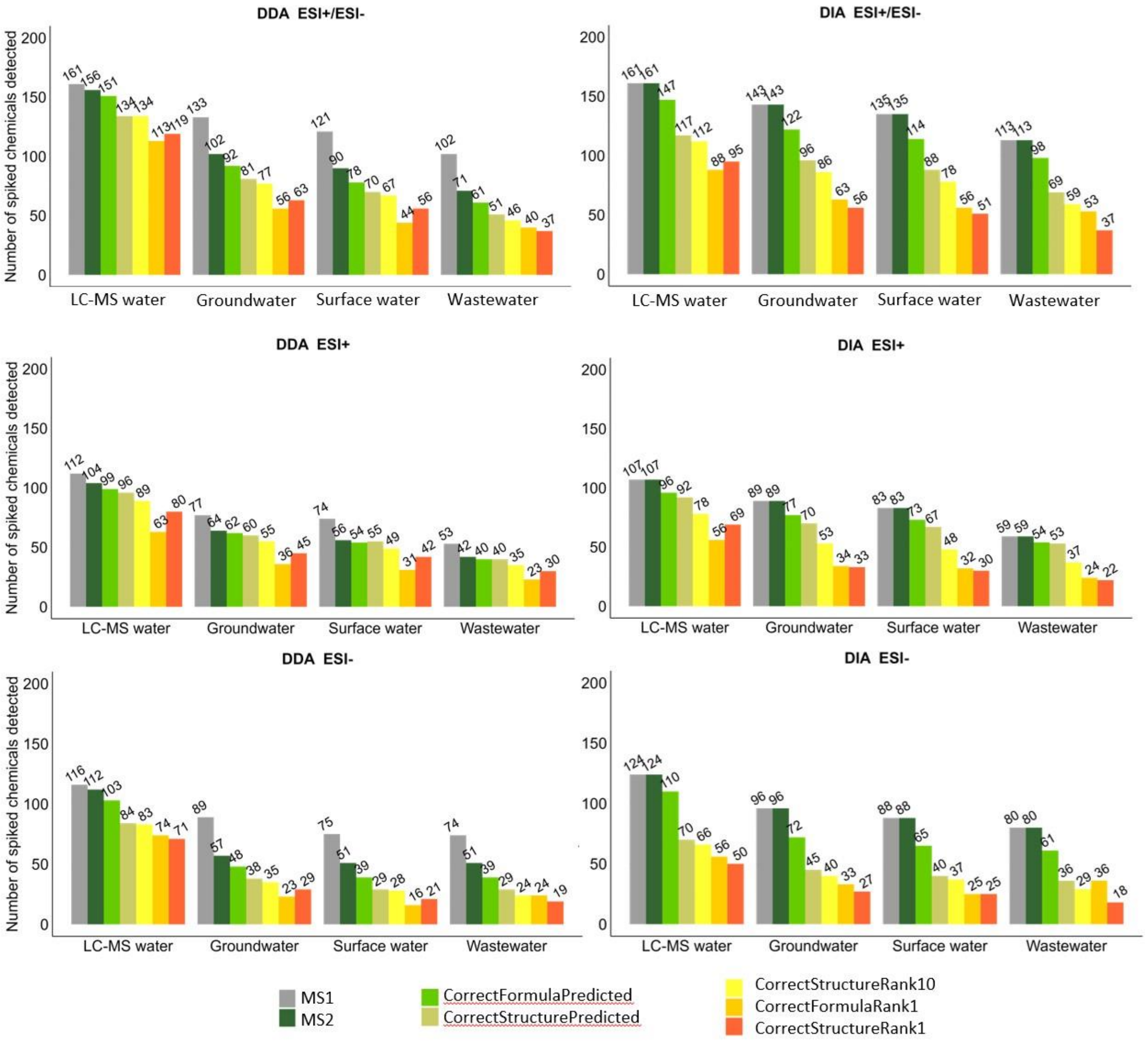

Figure S1. Number of spiked reference chemicals detected in full scan MS1, and with acquired MS2 spectrum, separated by MS2 acquisition method and water sample type. First row shows combined values for positive and negative ionization mode, second row only compounds detected in positive mode and third row only compounds detected in negative mode. The corresponding numbers of correct *in silico* formula and structural annotations are shown as CorrectFormulaPredicted (correct formula is predicted by SIRIUS), CorrectStructurePredicted (correct structure is predicted with SIRIUS), CorrectFormulaRank10 (correct structure is ranked 1-10), CorrectFormulaRank1 (correct formula is ranked highest), and CorrectStructureRank1 (correct structure is ranked highest).

Table S8. All the data for creating Figure 1 in the main article for DDA and DIA comparison (additional .csv file named Table_S8_DDA_DIA_spiked_comparison_all.csv)

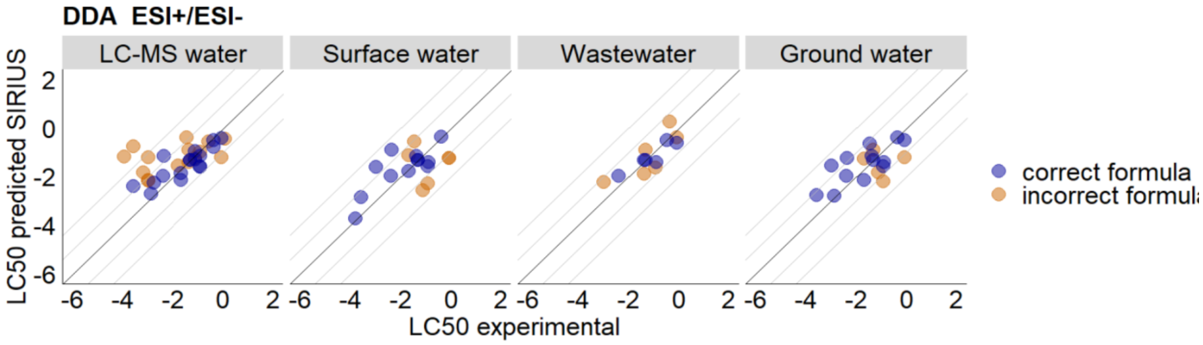

Figure S2. Comparison of MS2Tox predicted LC_{50} values from DDA MS^2 data versus experimental LC_{50}.

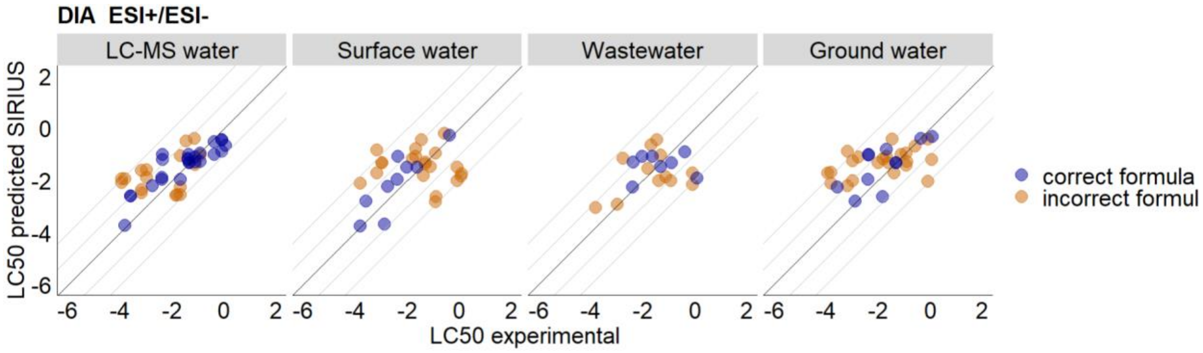

Figure S3. Comparison of MS2Tox predicted LC_{50} values from DIA MS^2 data versus experimental LC_{50}.

Table S9. Spiked chemicals toxicity predictions and confidence scores for Figure 2 in the main text. Table as an additional file named Table_S9_SpikedChemicals_AllToxConf.csv.

Table S10. Toxicity predictions and confidence scores for non-targeted data from water sample. Data for Figure 3 in the main text. Table as an additional file named Table_S10_MatrixFeatures_ToxConfidence.csv.

A) 4 fixed DIA windows: B) 5 DIA windows variable size:

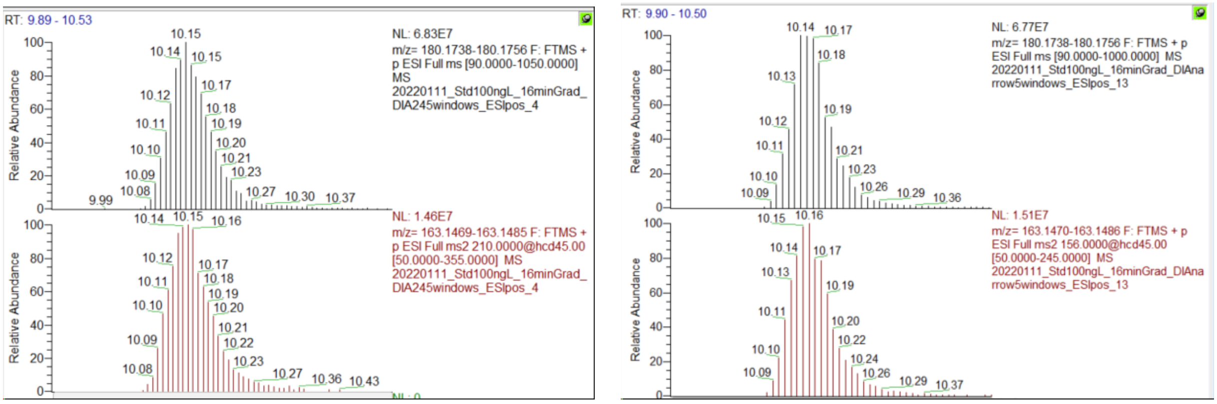

Figure S4. Chromatograms showing scans per peak of memantine (spiked to 100 ng/L in LC-MS water) with two DIA methods: A) 4 fixed DIA windows and B) 5 variable DIA windows. In black, MS1 precursor ion [M+H]+ m/z 180.1747 and in red most MS2 fragment m/z 163.1478. Show acceptable number of scans per peak with both DIA methods.

A) 4 fixed DIA windows: B) 5 DIA windows variable size:

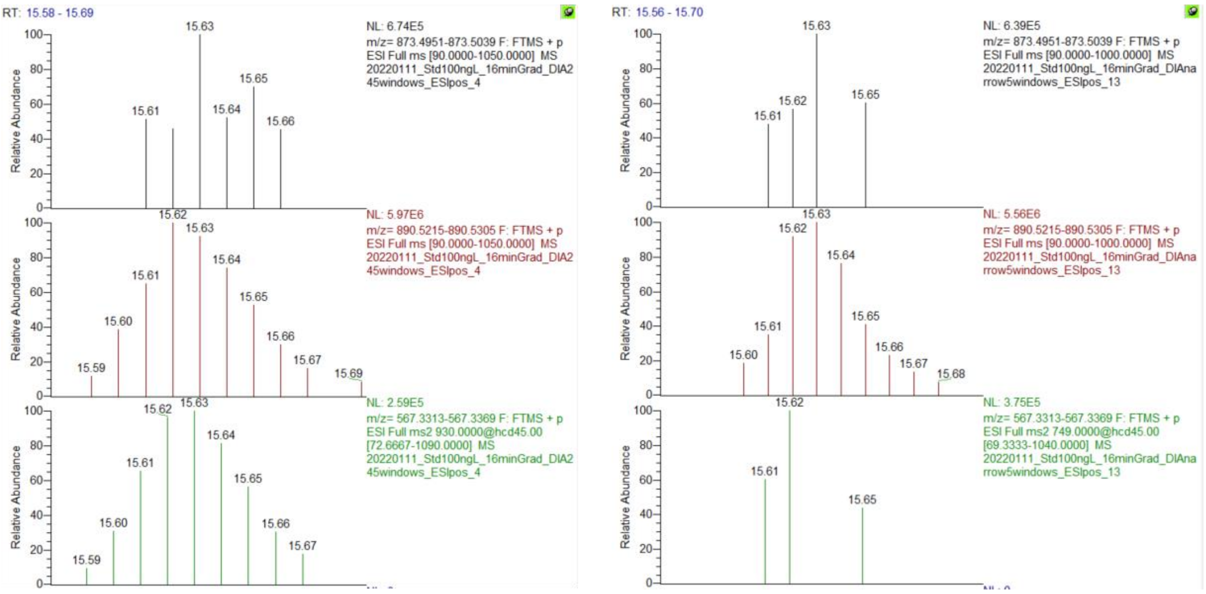

Figure S5. Chromatograms showing scans per peak of abamectin (spiked to 100 ng/L in LC-MS water) with two DIA methods: A) 4 fixed DIA windows and B) 5 variable DIA windows. n black, MS1 precursor ion [M+H]+ m/z 873.4995 and in red MS1 precursor ion [M+NH4]+ m/z 890.5260, in green MS2 fragment m/z 567.3341. Show loss of scans per peak when increasing number of acquisition windows.

References

1. Gago-Ferrero et al. (2018). Env. Sci. Technol. DOI: 10.1021/acs.est.7b06598
2. Golovko et al. (2020). Chemosphere. DOI: 10.1016/j.chemosphere.2020.127293
3. Li et al. (2016). Env. Sci. Technol. DOI: 10.1021/acs.est.5b06327
4. Li et al. (2018). Environ. Sci.: Processes Impacts. DOI: 10.1039/c7em00552k
5. Tröger et al. (2018). STOTEN. DOI: 10.1016/j.scitotenv.2018.01.277
6. Herzog & Maxe (2019). SGU-rapport 2019:17. Diarie-nr: 35-1157/2018
7. Carlström & Maxe (2019). SGU-rapport 2019:02. Diare-nr 35-782/2016
8. Malnes et al. (2022). Chemosphere. DOI: 10.1016/j.chemosphere.2022.133825
9. Schulze et al. (2019). Water Reasearch Vol 153. DOI: 10.1016/j.watres.2019.01.008
10. Arp & Hale (2022). ACS Environ. 6. DOI: 10.1021/acsenvironau.2c00024
11. European Commission: Water Framework Directive 2000/60/EC. https://environment.ec.europa.eu/topics/water/surface-water_en (visited 2022-12-21).
12. Figuiere et al. (2022). J. Hazard. Mat. DOI: 10.1016/j.jhazmat.2022.128302
13. Bonnefille et al. (2023). Soon published
14. Sdougkou et al. (2023). Soon published
15. Papazian et al. (2022). Commun Earth Environ 3. DOI: 10.1038/s43247-022-00365-1
